# Selection of single domain anti-transferrin receptor antibodies for blood-brain barrier transcytosis using a neurotensin based assay and histological assessment of target engagement in a mouse model of Alzheimer’s related amyloid-beta pathology

**DOI:** 10.1371/journal.pone.0276107

**Published:** 2022-10-18

**Authors:** Shiran Su, Thomas J. Esparza, David L. Brody

**Affiliations:** 1 National Institute of Neurological Disorders and Stroke, Bethesda, Maryland, United States of America; 2 Department of Biomedical Engineering, Washington University in St. Louis, St. Louis, Missouri, United States of America; 3 Center for Neuroscience and Regenerative Medicine, Henry M. Jackson Foundation for the Advancement of Military Medicine, Bethesda, Maryland, United States of America; 4 Henry M. Jackson Foundation for the Advancement of Military Medicine, Inc, Bethesda, Maryland, United States of America; 5 Department of Neurology, Uniformed Services University of the Health Sciences, Bethesda, Maryland, United States of America; Eötvös Loránd Research Network Biological Research Centre, HUNGARY

## Abstract

The blood-brain barrier (BBB) presents a major obstacle in developing specific diagnostic imaging agents for many neurological disorders. In this study we aimed to generate single domain anti-mouse transferrin receptor antibodies (anti-mTfR VHHs) to mediate BBB transcytosis as components of novel MRI molecular contrast imaging agents. Anti-mTfR VHHs were produced by immunizing a llama with mTfR, generation of a VHH phage display library, immunopanning, and *in vitro* characterization of candidates. Site directed mutagenesis was used to generate additional variants. VHH fusions with neurotensin (NT) allowed rapid, hypothermia-based screening for VHH-mediated BBB transcytosis in wild-type mice. One anti-mTfR VHH variant was fused with an anti-amyloid-beta (Aβ) VHH dimer and labeled with fluorescent dye for direct assessment of *in vivo* target engagement in a mouse model of AD-related Aβ plaque pathology. An anti-mTfR VHH called M1 and variants had binding affinities to mTfR of <1nM to 1.52nM. The affinity of the VHH binding to mTfR correlated with the efficiency of the VHH-NT induced hypothermia effects after intravenous injection of 600 nmol/kg body weight, ranging from undetectable for nonbinding mutants to -6°C for the best mutants. The anti-mTfR VHH variant M1_P96H_ with the strongest hypothermia effect was fused to the anti-Aβ VHH dimer and labeled with Alexa647; the dye-labeled VHH fusion construct still bound both mTfR and Aβ plaques at concentrations as low as 0.22 nM. However, after intravenous injection at 600 nmol/kg body weight into APP/PS1 transgenic mice, there was no detectible labeling of plaques above control levels. Thus, NT-induced hypothermia did not correlate with direct target engagement in cortex, likely because the concentration required for NT-induced hypothermia was lower than the concentration required to produce *in situ* labeling. These findings reveal an important dissociation between NT-induced hypothermia, presumably mediated by hypothalamus, and direct engagement with Aβ-plaques in cortex. Additional methods to assess anti-mTfR VHH BBB transcytosis will need to be developed for anti-mTfR VHH screening and the development of novel MRI molecular contrast agents.

## Introduction

Alzheimer’s disease (AD) is one of the most important causes of dementia in the elderly [1]. About 6.2 million Americans are living with AD, and it’s predicted that the number will increase to 13.8 million by 2050 [2]. With no effective therapies to cure or inhibit significant AD symptom progression [3], AD severely decreases patients’ quality of life and creates an enormous burden on the health care system and society [1, 4]. Currently, clinical AD diagnosis is based on cognition and the relative impact of impairments on daily activities [5]. However, multiple neurodegenerative and vascular pathologies can coexist and produce cognitive and behavioral symptoms which could overlap with each other [6]. This makes it difficult to accurately identify pathology based solely on clinical symptoms. The accuracy of clinical diagnosis of AD at the National Institute of Aging and National Institute of Aging sponsored AD centers varies depending on the clinical and neuropathologic criteria used [7]. The sensitivity of AD diagnosis ranges from 70.9% to 87.3%, whereas specificity ranges from 44.3% to 70.8%, which need to be improved. AD has a very long prodrome stage before clinically observable symptoms [8]. Early diagnosis is preferred to help with early intervention and the development of preventive therapeutics to slow AD development [5]. Detection of patients in the preclinical stages of AD can also help to provide diagnostic information, monitor disease progression, and even monitor the effect of newly developed treatment methods. Imaging methods including MRI and PET have been developed to aid the diagnosis of AD [9, 10]. However, PET imaging for AD provides limited resolution and requires radiation exposure. Structural MRI lacks specificity and does not allow direct visualization of Aβ or tau, the histological hallmarks of AD. There is an unmet need for developing methods like molecular contrast MRI which have better resolution than PET and better specificity than structural MRI imaging.

The blood-brain barrier (BBB) represents a significant obstacle in delivering diagnostic and therapeutic agents to the central nervous system (CNS), preventing uptake of more than 98% of potential neurotherapeutics to brain [11–14]. The BBB consists of endothelial cells held together by tight junctions which hinder paracellular passage. Most molecules do not transfer from blood to brain through the BBB, which protects the brain from toxicity and maintains brain homeostasis. Several methods have been developed to improve the transport of diagnostic and therapeutic agents into the CNS. For instance, the BBB may be temporarily opened by administration of hypertonic agents or focused ultrasound [15, 16]. Alternatively, very high doses of an agent can be given so that even if a small fraction enters the brain the desired effect will be achieved. In some cases, direct injection of agents into the cerebrospinal fluid can be employed [14]. However, these methods are invasive and have a risk of causing infection, toxicity, and neurological dysfunction [13, 14].

BBB crossing based on receptor mediated transcytosis (RMT) is potentially especially promising. The use of protein shuttles has the potential to facilitate the transport of therapeutic agents across the BBB using specific endogenous receptor systems. Candidate receptor systems including transferrin receptors (TfR), low-density lipoprotein receptors, insulin receptors and neuropeptide receptors are highly expressed on the BBB where they mediate RMT [13]. Among the different receptors, TfR has been widely used for transporting macromolecules across the BBB [17, 18]. Based on the study of Yu et al., there is a nonlinear relationship between an antibody’s affinity for TfR and its uptake in brain. At tracer doses, antibodies with higher affinity to TfR have higher uptake into the brain, while at therapeutic doses, antibodies with lower affinity to brain have higher uptake into the brain [19]. The effect of TfR affinity on brain uptake has been confirmed by the study of Wiley et al [20]. Transferrin conjugated gold nanoparticles with high avidity to TfR remain strongly attached to brain endothelial cells and reduced accumulation in brain parenchyma compared with nanoparticles with lower avidity to TfR [20]. Thus, to achieve optimal brain uptake, it is important to optimize anti-TfR concentration and antibody affinity to TfR. Jefferies et al. identified a monoclonal antibody OX-26 specific for transferrin receptors [21]. This antibody was tested and was confirmed to be able to facilitate TfR-mediated transcytosis across BBB [22]. Kissel et al. produced monoclonal antibody 8D3 which recognizes murine transferrin receptor [23]. Yu et al. generated a bispecific antibody that binds to TfR for transcytosis and also to the enzyme β-secretase for inhibiting Aβ production [19]. Hultqvist et al. attached a single chain variable fragment against TfR to the anti-amyloid-beta protofibril recombinant monoclonal antibody RmAb158. The anti-TfR single chain variable fragment increased brain uptake of the antibody by 80-fold [24]. However, most of the existing anti-TfR antibodies have relatively large size, especially when conjugated to additional components for RMT or other payloads such as imaging contrast agents. Previous research found that reducing the size of nanoparticles helped to improve blood brain barrier transcytosis [25] Keeping the size of each component small is important since the ultimate goal of this line of investigation is to develop multicomponent brain MRI molecular contrast agents. Also, it is expensive to engineer and synthesize monoclonal antibodies, which are typically produced in mammalian cell culture. Thus, there is an unmet need for smaller, less expensive, and easier to engineer system for BBB transcytosis.

Camelids produce functional antibodies devoid of light chains called heavy chain-only antibodies (HCAbs) [26, 27]. HCAbs recognize their cognate antigens by one single domain, the variable domain (VHH). The VHH in isolation is very small compared with other antibodies. The molecular weights of VHHs are typically ~15kDa, which is about 1/10 of the molecular weight of a conventional IgG and about half the molecular weight of a single chain variable fragment (Scfv) [27, 28]. VHHs have affinities at the same order of magnitude as conventional IgGs, often in the nanomolar or subnanomolar range [29]. Because of their small size, VHHs can also bind to epitopes not recognized by conventional antibodies and can have better tissue penetration capacities [30, 31]. The factors governing VHH immunogenicity are similar to those for conventional antibodies and VHHs have been demonstrated to have low immunogenicity risk profile [32, 33]. Furthermore, because VHHs are devoid of Fc domains, they do not induce antibody-dependent cellular cytotoxicity (ADCC) or complement-dependent cytotoxicity (CDC) [34, 35]. Caplacizumab was the first VHH approved by FDA for treatment of acquired thrombotic thrombocytopenic purpura in humans [36]. There are several more VHHs which are in clinical trials, with safety profiles similar to other antibody therapeutics in humans [37, 38]. Importantly, VHHs have been found that can facilitate BBB penetration and allow brain target binding [39–44]. The use of VHHs for BBB transcytosis and target engagement is promising. Specifically, Stanimirovic et al. identified an insulin-like growth factor 1 receptor binding VHH which crosses the BBB by RMT [39, 45, 46]. Danis et al. identified and optimized VHHs to mitigate brain accumulation of pathological tau in a tauopathy mouse model [40]. Dupré et al., identified VHHs which could be used to detect tau in transgenic mice brain tissues [41]. However, the extent to which VHHs that engage in RMT using the TfR can carry diagnostic and therapeutic payloads across the BBB still has not been fully assessed. Here we tested the hypothesis that VHHs that bind to TfR and cross the BBB through RMT in mice could be coupled with additional VHHs that bind to amyloid plaques as a proof of concept for a platform which could be generalized to other neurological diseases.

Current methods (ELISA or radioisotope detection) to evaluate BBB crossing require substantial resources and can be time-consuming [19, 47, 48]. An efficient way to screen for BBB crossing *in vivo* would be helpful since *in vitro* models may not be fully predictive of *in vivo* results [49]. Neurotensin (NT) is a 13 amino acid peptide first identified by Carraway and Leeman in 1973 from bovine hypothalamic extracts [50]. NT is expressed in CNS as well as in peripheral tissues, mostly in the gastrointestinal tract. NT is involved in regulating appetite, nociception, and thermoregulation in the CNS, and alters nutrient absorption, gastrointestinal motility, and secretion in the peripheral gastrointestinal tract [51]. It was found that NT induces rapid and transient hypothermia in mice and rats when injected to CNS [52, 53]. The hypothermia was likely to be due to effects in hypothalamus. Young & Kuhar found that NT receptors had moderate to high densities in hypothalamus [54]. Injection of NT to medial, lateral preoptic area and anterior parts of hypothalamus induced the hypothermia effect [54]. Meanwhile, intravenously injected NT does not typically cause hypothermia [55]. On the other hand, when NT-conjugated mouse TfR (mTfR)-binding VHHs were injected intravenously to mice, the NT-mTfR VHH conjugates induced hypothermia in mice, presumably because they get across the BBB and bind to NT receptors in hypothalamus [44, 47]. These properties of the NT system make it an apparently attractive assay platform for rapid testing of VHH BBB transcytosis.

**Fig 1:**
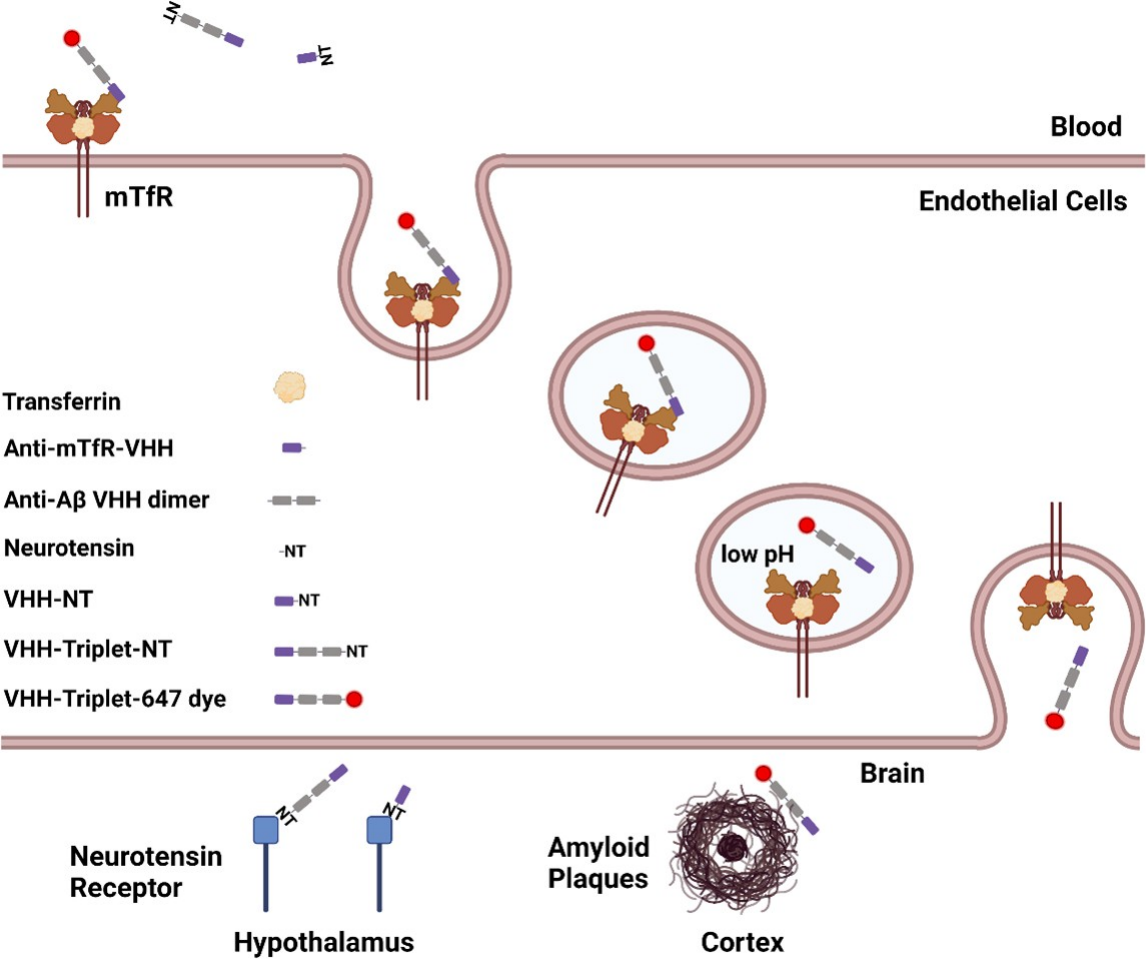
Schematic of the modular system for VHH-mediated BBB transcytosis and target engagement. Module 1: anti-mTfR VHH for RMT across BBB. Module 2: neurotensin peptide (NT) for rapid assessment of *in vivo* target engagement via measurement of hypothermia. Module 3: anti-Aβ VHH dimer for disease-relevant target engagement in brain parenchyma. Module 4: 647 dye for visualization of VHH constructs in situ. The anti-mTfR VHH was combined with NT for anti-mTfR VHH screening through monitoring hypothermia effect. The anti-mTfR VHH were combined with VHH dimer and NT or 647 to test the effect of anti-mTfR VHH together with other components of the module. This schematic figure also shows the TfR mediated transcytosis across BBB. The anti-mTfR VHH conjugates binds to TfR on the endothelial cells which comprise a major portion of the BBB. Then the TfR-VHH complex is endocytosed across the endothelial cells in the endosomes. VHHs dissociate from the TfR-VHH complex with decreased pH level in endosome and are released on the brain side of the BBB. There were two different targets in the brain parenchyma in this study, the NT receptor in hypothalamus and amyloid plaques in cortex and hippocampus. (Figure generated using BioRender.com).

Here, we independently generated additional mTfR-binding VHHs and used a similar NT based modular system to screen these VHHs for mTfR-mediated BBB transcytosis (**Fig 1**). We hypothesized that this modular screening system can be used to identify and optimize anti-mTfR VHHs for BBB transcytosis and brain target binding.

This screening system includes four modules. The first module is an anti-mTfR VHH for RMT across the BBB; the second module is the neurotensin peptide for assessment of target engagement *in vivo* through measurement of hypothermia. The third module is a dimer of anti -Aβ VHHs for disease-relevant brain target engagement. The fourth module is a fluorescence dye for visualization of VHH constructs in situ. We tested different VHH variants and found one variant which showed good BBB penetration based on this screening system. The VHH variant with the best BBB transcytosis ability fused to a tandem VHH dimer called Nb3-Nb3 which binds to amyloid plaques in brain parenchyma. This VHH triplet was conjugated to a fluorescent dye and post-mortem confocal microscopy was performed to directly evaluate brain target engagement.

## Materials and methods

### Immunization of llama with mouse transferrin receptor

A single adult male llama (*Lama glama*) was immunized under contract agreement through Triple J Farms (Kent Laboratories, Bellingham, WA) following the method previously described [56]. Briefly, subcutaneous injections of 100 μg ectodomain (Cys89-Phe763) mTfR (50741-M07H, SinoBiological) (synthesized in HEK293 cells and went through glycosylation) were performed with protein emulsified with complete Freund’s adjuvant on day 0, followed by additional 100 μg immunizations emulsified with incomplete Freund’s adjuvant on days 14, 28, and 42. On day 49, peripheral blood was drawn for peripheral blood mononuclear cell (PBMC) isolation. Triple J Farms operates under established National Institutes of Health Office of Laboratory Animal Welfare Assurance certification number A4335-01 and United States Department of Agriculture registration number 91-R-0054.

### Generation of VHH immune phage display library

The generation of an immune phage display library and isolation of mTfR binding VHH clones was performed using the methods previously described [56]. Briefly, total RNA extracted from PBMCs was used for synthesis of first-strand complimentary DNA (cDNA) using the SuperScript IV First-Strand Synthesis kit (#1891050, Invitrogen). The heavy-chain variable domain was then amplified from the cDNA using Q5 high-fidelity DNA polymerase (New England Biolabs) with the described primers (CALL001: 5′-GTCCTGGCTGCTCTTCTACAAGG-3′ and CALL002: 5′-GGTACGTGCTGTTGAACTGTTCC-3’). The heavy-chain specific amplicon was isolated using electrophoresis with low-melting point agarose extraction with the QIAquick Gel Extraction kit (Qiagen). A secondary amplification was performed using a modification of the primers (VHH-Esp-For: 5′-CCGGCCATGGCTGATGTGCAGCTGCAGGAGTCTGGRGGAGG-3′ and VHH-Esp-Rev: 5′-GTGCGGCCGCTGAGGAGACGGTGACCTGGG T-3′) used by Pardon et al. to facilitate cloning into the phagemid pHEN2 [57]. The amplified sequences were cleaved with the restriction endonucleases NcoI and NotI (New England Biolabs) and subsequently ligated into compatibly cleaved pHEN2 phagemid at a 3:1 (insert:phagemid) ratio overnight at 16°C followed by purification. The resulting ligation mixture was electroporated into TG-1 phage-display competent cells (#60502–1, Lucigen) and plated onto 2xYT agar containing 100 μg/mL carbenicillin and 2% (w/v) glucose at 37°C overnight. The resulting library contained > 10^7^ independent clones. Phage was produced for screening using the M13KO7 helper phage (#18311019, Invitrogen) followed by precipitation by addition of one-fifth volume 20% polyethylene glycol 6000 / 2.5 M sodium chloride solution on ice and centrifugation to purify the phage particles.

### Immunopanning and clone screening

Selection of mTfR specific VHH was performed using direct binding of phage to immobilize mTfR. Standard radioimmunoassay (RIA) tubes were coated with 500 μL mTfR solution at 5 μg/mL in sodium carbonate buffer, pH 9.6 overnight at 4°C. The coating solution was removed, and the RIA tube filled with a 2% (w/v) non-specific blocking solution (bovine serum albumin or nonfat dry milk) in 1x Phosphate Buffered Saline (PBS). Amplified phage (~10^11^ phage) was mixed with blocking solution to a final volume of 500 μL and then transferred into the RIA tube to allow for association at room temperature and 600 rpm mixing. The RIA tube was then washed 20 times with 1x PBS and then the bound phage eluted with 100 mM triethylamine solution for 20 mins. The eluted phage solution was neutralized with 1:10 volume 1 M Tris-HCl, pH 8.0. The eluted phage was amplified in TG-1 cells and a second round of immunopanning was performed.

Following the second round of immunopanning, individual colonies were selected and cultured in 96-well blocks containing 2xYT containing carbenicillin at 37°C with 300 rpm shaking for 4–6 hours. Expression of VHH was induced by addition of isopropyl-beta-D-thiogalactoside (IPTG) to a final concentration of 1 mM and incubation overnight at 37°C. The culture blocks were centrifuged to pellet the cells and frozen at -80°C for 1 hour following removal of the culture supernatant. The culture block was then equilibrated to room temperature and 500 μL 1xPBS added to each well followed by shaking at 1500 rpm to resuspend the cell pellets and allow for release of VHH from the cells. The culture block was centrifuged for 20 min at 2000xg. Nunc Maxisorp plates were coated with mTfR at 1 μg/mL as described above and blocked with 1% bovine serum albumin (BSA). The clarified VHH supernatants were incubated on the mTfR plates for 1 hour at room temperature. The assay plate was washed and peroxidase conjugated goat anti-alpaca VHH domain specific antibody (#128-035-232, Jackson ImmunoResearch) at 0.8 μg/mL was transferred to the plate and incubated for 1 hour at room temperature. Following a final wash, the assay was developed by addition of tetramethylbenzidine (#T5569, Sigma-Aldrich) and absorbance was measured at 650 nm on a Biotek Synergy 2 plate reader. Clones with absorbance values greater than two standard deviations above background were considered of interest and subsequently sequenced.

### Amyloid beta specific VHH production

Paraschiv et al. previously reported the isolation of amyloid beta binding VHH clones [58]. We selected the sequence for the named Nb3 clone for use in this study. The Nb3 amino acid sequence was imported into SnapGene software (GSL Biotech LLC) and reverse translation performed using preferred codon usage for expression in *E*. *coli*. Additional sequence, including a (Gly-Gly-Gly-Ser)_3_ between VHH domains, was incorporated for cloning into pHEN2 as a tandem dimer as part of the heterotrimer clones synthesized with TfR binders. To reduce the potential for recombination events, the DNA sequence was manually curated to adjust the codon usage and reduce the frequency of repetitive sequence within the Nb3-Nb3 dimer. The affinity of the Nb3-Nb3 dimer was measured using bio-layer interferometry as described below.

### Bio-layer interferometry (BLI) assessment of VHH binding kinetics

The binding kinetics of the selected VHH clone against mTfR and Aβ was assessed by BLI. For measurements of mTfR kinetics, biotinylated VHH was diluted into assay buffer at 1 μg/mL and immobilized onto streptavidin coated biosensors (#18–5019, Sartorius) to a minimum response value of 1 nm on the Octet Red96 System (Sartorius). For measurements of amyloid beta, beta-amyloid(1–40)-Lys(biotin-LC) (AS-23517, Anaspec), was diluted into assay buffer at 1 μg/mL and immobilized onto streptavidin coated biosensors (#18–5019, Sartorius) to a minimum response value of 1 nm on the Octet Red96 System (Sartorius). Purified mTfR or VHH clones were diluted into assay buffer at the specified concentrations. The immobilized antigen biosensors were allowed to associate at 37C° followed by dissociation in the baseline buffer well location. All assays included a background correction condition to allow for sensor normalization. The ForteBio Data Analysis suite was used to normalize the association curves following background subtraction and Savitzky-Golay filtering. Curve fitting was applied using global fitting of the sensor data and a steady state analysis calculated to determine the association and dissociation constants. All assay steps were prepared in Greiner 96-well plates (#655209) in a volume of 300 μL. Assay buffer was defined as 0.1% BSA (w/v) in 1xPBS.

### Synthesis and expression of VHH constructs

Based on the methods described in **Immunopanning and clone screening** and **BLI assessment of VHH binding kinetics** sections, we synthesized several neurotensin-fused VHH monomers with different binding affinities to mouse transferrin receptor. In addition, we produced single polypeptide VHH heterotrimers that consisted of M1_P96H_, a (Gly-Gly-Gly-Ser)_3_ linker, Nb3, a (Gly-Gly-Gly-Ser)_3_ linker, and Nb3 using the method described in **Amyloid beta specific VHH production**. The VHH dimer Nb3-Nb3 binds to amyloid plaques in brain parenchymal of APP/PS1 mice. These constructs were termed “M1_WT_-NT”, “M1_P96H_-NT”, “M1_AA_-NT”, “M1_R100dH_-NT” and “M1_P96H_-triplet”. The VHH naming convention for P96H and R100dH are based on the Kabat nomenclature and refer to the specific amino acid residue modification positions within the VHH sequence [59]. Another neurotensin fusion with a VHH generated against human TfR, which does not bind mouse TfR called “H1-NT,” was used as control. All VHH constructs were designed using SnapGene Software and synthesized (Twist Bioscience) with corresponding restriction endonuclease sites for direct cloning into pHEN2. Sequence confirmed pHEN2 clones with the various constructs were transferred into BL21(DE3) competent *E*. *coli* cells (C2527I, New England Biolabs). Transformed cells were grown in terrific broth medium containing carbenicillin at 37°C and 300rpm shaking in baffled flasks. Once the culture density reached an optical density equal to 0.6, IPTG was added to a final concentration of 1mM to induce protein expression. For monomer VHH expression the post-induction incubation temperature was maintained at 37C but reduced to 30C for the M1_P96H_-triplet. Following overnight expression, cells were pelleted by centrifugation and VHHs were extracted through osmotic shock and recovery of the periplasmic fraction [56, 60]. Clarified periplasmic fraction was purified using HisPur™ Ni-NTA Resin (88222, Thermo Fisher Scientific) column chromatography. The eluted VHH proteins were further purified by size-exclusion chromatography (SEC) over a Superdex75 10/300 column on an AKTA Pure (Cytiva). Protein purity was assessed by SDS-PAGE on a 10% Bis-Tris MES acrylamide gel and found to be >95% pure.

M1_P96H_-triplets and H1-triplets were fluorescently labeled with using Alexa Fluor^TM^ 647 succinimidyl ester dye to allow fluorescence confocal microscopy of tissue sections. The triplets were incubated with the succinimidyl ester dye for 1 hour in 50mM sodium carbonate buffer, pH 9.6 and purified by desalting using a 5mL HiTrap desalting column in-line with the AKTA Pure. The binding fidelity of the VHH heterotriplet was assessed by ELISA against mTfR. The fluorescence dye labeled M1_P96H_-triplet and H1-triplet were named “M1_P96H_-triplet-647” and “H1-triplet-647”.

### Endotoxin removal

Removal of contaminating endotoxin was achieved using High-Capacity Endotoxin Removal Resin (88270, Pierce) from the VHH preparations. A volume of 0.25ml endotoxin removal resin was added to 1ml of VHH sample. The VHH and resin were mixed for 2hr at room temperature to allow for absorption of endotoxin. The mixture was centrifuged to pellet the resin and the supernatant collected. A volume of 0.25ml fresh endotoxin removal resin was added to the first-pass solution and mixed for another 2hr at room temperature. Following a final centrifugation to pellet the resin, the solution was collected for endotoxin level testing. Endotoxin levels were measured using the Chromagenic Endotoxin Quant kit (A39552, Pierce) according to the manufacturers protocol to ensure a level of endotoxin <0.5 endotoxin units/mg of total protein.

### Binding assessment of histidine mutations by ELISA

To determine the effect on histidine mutation introduction, an ELISA was performed with the post VHH binding wash buffer at normal and reduced pH. Nunc Maxisorp plates were coated with mTfR at 1 μg/mL in 50mM sodium carbonate, pH 9.6 overnight at 4°C. The plates were blocked with 1% BSA in 1xPBS buffer and then triplicate dilutions of each VHH prepared in 0.5% BSA in 1xPBS buffer and transferred to the plate. Binding at room temperature for 2 hours was followed by three cycles of 5-minute washes with either 1xPBS, pH 7.2 or 1xPBS, pH 5.5. Following the pH dependent wash step, the bound VHH was detected with peroxidase conjugated goat anti-alpaca VHH domain specific antibody (#128-035-232, Jackson ImmunoResearch) at 0.8 μg/mL incubated for 1 hour at room temperature. Following a final wash, the assay was developed by addition of tetramethylbenzidine (#T5569, Sigma-Aldrich), the reaction was terminated by addition of 50 μL 1M hydrochloric acid and absorbance was measured at 450 nm on a Biotek Synergy 2 plate reader.

### Animals

All animal experiments were conducted under protocol approved by the National Institute of Neurological Disorders and Stroke (NINDS)/ National Institute on Deafness and Other Communication Disorders (NIDCD) Animal Care and Use Committee in the National Institutes of Health (NIH) Clinical Center (Protocol Number: 1406–21). C57BL/6J female mice were purchased from Jackson labs at 6–7 weeks of age and used at 7–12 weeks of age. Fifteen mice were divided into five groups with three mice in each group to test the five VHH-NT fusions. Anesthetized mice were injected via tail vein with VHH-NT fusions: M1_WT_-NT, M1_P96H_-NT, M1_AA_-NT, M1_R100dH_-NT and H1-NT for screening. Experiments were performed at the same time each day. To confirm the result from the five VHH-NT fusions, a blinded replication experiment was performed with additional fifteen mice randomized using a random number generator into five groups with three mice in each group. Six mice were randomly assigned into two groups to test the ability of VHH triplet-NT to get across the BBB after fused to anti-Aβ dimer Nb3-Nb3. Three mice were injected with M1_P96H_-Triplet-NT and three mice were injected with H1-Triplet-NT.

APP_SWE_/PSEN1dE9 (MMRRC Strain #034832) positive transgenic mice were purchased from Jackson labs [61, 62], maintained on a hybrid (C57BL/6xC3H) background, and raised under protocols approved by the National Institute of Neurological Disorders and Stroke (NINDS)/ National Institute on Deafness and Other Communication Disorders (NIDCD) Animal Care and Use Committee in the National Institutes of Health (NIH) Clinical Center. Ten transgenic mice ages between 13–15 months, eight males and two females, were randomly assigned to two groups. Five mice were injected with M1_P96H_-triplet-647 and five mice were injected with H1-triplet-647.

### Injection of VHH-NTs and VHH-triplet-647s

Mice were anesthetized with 60% oxygen/ 40% medical air gas mixture containing 5% isoflurane in an induction box. After a stable anesthesia plane was established, mice were maintained at 1.5–2% isoflurane level. Artificial tears ointment was applied to prevent eye injury due to drying. Mice were placed on an electrical heating pad to maintain body temperature at 37C°. The VHH-NTs, M1_WT_-NT, M1_P96H_-NT, M1_AA_-NT, M1_R100dH_-NT and H1-NT were injected into wild-type C57BL/6J mice through the tail vein using a 30 Gauge needle in a single bolus at a dose of 600nmol/kg body weight in 1xPBS. The VHH-triplet-647s, M1_P96H_-triplet-647 and H1-triplet-647, were injected intravenously through single bolus injection at a dose of 1000nmol/kg body weight. Mice were maintained under anesthesia for approximately 2–3 minutes. Following the procedure, the mice were allowed to recover on a heating pad until fully ambulatory and then returned to their home cage with immediate access to food and water.

### Temperature measurement

Infrared thermometry was used for temperature measurement using an infrared thermometer (Model# 62 MAX+, Fluke). Abdominal fur was removed by application of topical depilatory cream prior to temperature measurement for precise data collection. Baseline temperature was measured three times before intravenous (IV) injection with a time interval of 30min between measurements. Then, mouse temperatures were measured at the time intervals of 30min, 1hr, 1.5hr, 2hr, 2.5hr, 3hr, 3.5hr, 4hr, 4.5hr and 5hr after injection. All temperature measurements were performed by investigators blinded to the identity of the injected VHH sample. Mice were briefly anesthetized (<20 seconds) with isoflurane for each temperature measurement. In preliminary experiments we confirmed that this brief anesthesia did not affect temperature.

### Confocal microscopy of mice brains injected with triplet-647

Two hours post IV injection mice were sacrificed under isoflurane anesthesia by cardiac perfusion with 1X PBS + Heparin (10 units Heparin per milliliter 1X PBS). Following perfusion, mice were decapitated using a pair of sharp surgical shears and the brain was carefully excised from the cranium. Brains were fixed in 4% PFA for 24hrs then equilibrated in 30% sucrose for 48 hours. Then, mouse brains were sectioned at 50 μm thickness using a freezing sliding microtome. Staining was performed to visualize amyloid plaques using the Congo Red derivative X34 [63]. Tissue was rinsed with 1xPBS two times. Then, tissues were incubated in 40%EtOH/60%PBS at pH10 containing 10 μM X34 (SML1954-5MG, Sigma) for 10min. After X34 incubation, tissue was rinsed with milliQ water five times then differentiated in 40%EtOH/60%PBS at pH10 for 2min. After differentiation, the tissue was rinsed with miliQ water for 10min then mounted onto positively charged slides (EF15978Z, Daigger®). The mounted tissue sections were allowed to dry overnight at room temperature and cover slipped using fluoromount-G (00-4958-02, Invitrogen™).

To objectively evaluate the effect of intravenous injection of M1_P96H_-Triplet-647 and H1-Triplet-647 on APP/PS1 mice, brain sections at around 2.5mm posterior to bregma were selected for confocal fluorescence imaging. The brain sections were equally divided into 10 parts in both horizontal (x) and vertical (y) directions (**S1 Fig**). X and y coordinates were randomly selected using by random numbers from 1 to 10. When (x, y) coordinates fell onto a cortical area, stacks of images were taken which covered 16μm depth (z direction) starting from the top of the brain section. When (x, y) coordinates fell outside of the cortex, the microscope was moved to the next randomized (x, y) coordinates without taking images. Brain sections were imaged using a Zeiss LSM 510 microscope. Stacks of images were acquired with eight images per stack and 2μm optical thickness at 20x magnification using a Zeiss Plan-Apochromat 20x/0.8NA lens. Images were acquired using laser wavelength at 633nm to visualize the VHH triplets conjugated with 647 dye and 405 nm wavelength laser to visualize the X-34 dye. For 647 channel images, laser at wavelength 633nm was used with a LP650 filter and laser power set to be at transmission 100%. For X34 channel images, laser at wavelength 405nm was used with a BP420-480 filter and laser power set to be at transmission 15%. The images were exported in lsm file format for image analysis using ImageJ.

### Fluorescent confocal microscopy—Naïve brain as controls

Naïve APP/PS1 positive mouse brain sections stained only with X-34 for fluorescence confocal microscopy were used as a negative control. Naïve APP/PS1 positive mouse brain sections stained with both X34 and *ex vivo* M1_P96H_-Triplet-647 for fluorescence confocal microscopy were used as a positive control. For *ex vivo* M1_P96H_-Triplet-647 staining, naïve brains were sectioned and washed in 1XPBS. Sections were incubated in 3% normal donkey serum for 30min to block non-specific binding. After blocking, tissue sections were incubated overnight at 4C° in M1_P96H_-Triplet-647 at a concentration of 10ug/ml in blocking solution. Tissues were then washed in 1xPBS three times followed by X34 staining as described.

### Automated image analysis

To quantitatively analyze the confocal microscope images, thresholding and particle analysis were performed to remove background signals and isolate target structures of interest. The percent area of X34 stained amyloid plaques was quantified with thresholding at (0, 30) followed by Particle analysis. To remove low signal background, 647 channel image thresholding was set at 0–1000. Then the 647 channel images was analyzed with particle analysis. The parameters for particle analysis were set to be 0–1000 pixels for size and 0.1–1.0 for circularity. The parameters for thresholding and particle analysis were determined through testing of different parameters to capture the qualitative morphology of amyloid plaques as assessed by experienced investigators. Amyloid plaque levels were represented as % area of X34 coverage and VHH triplet-647 entry into the brain parenchyma was represented as % area of Alexa 647 dye coverage in the areas with X34 staining. Because there is mouse-to-mouse variability in plaque size and X34 staining intensity, we analyzed the ratio of the Triplet-647 coverage to X34 coverage.

### Statistical analysis

Unpaired student t-test were performed to evaluate the differences between the transcytosis ability of M1_P96H_-triplet-647 and H1-triplet-647 to amyloid plaques in APP/PS1 transgenic mice brains. Graphs were created using Prism. Sample sizes were based on availability of transgenic mice and previous experiments. No formal power calculations were performed.

## Results

### Endotoxin removal

VHHs contamination by endotoxins is a byproduct of expression in *E*. *Coli* cells [64]. Endotoxin can induce systemic inflammation and cause disruptive BBB changes [65]. It was found that the excess endotoxin level in VHH-NT fusions can cause additional hypothermia (**S2 Fig**). To avoid potential hypothermia effects caused by endotoxin, endotoxin was removed after purification. After endotoxin removal, the level of endotoxin level in VHHs was less than 0.1 endotoxin unit/ml (EU/ml) within normal range [66].

### Anti-mTfR VHH variant screening based on binding affinity measurement

VHH monomer-NT (M1-NT and H1-NT) and VHH-heterotrimer-NT (M1_P96H_-triplet-NT) had characteristics consistent with expectation. **S3 and S4 Figs** showed the SDS-PAGE gel and exemplar size-exclusion column results of the VHH monomer-NT and VHH-heterotrimer-NT after purification. VHH monomer-NTs had size around 14kDa (**S3 Fig**) and VHH heterotrimer-NTs had size between 38-49kDa (**S4 Fig**).

Histidine mutations could potentially impart pH dependence of binding [67]. Histidine protonation at lower pH-values can increase dissociation rate of antibodies to their receptors. Maeda et al. found that the dissociation rate was less rapid in the intracellular acidic compartments once histidine was deleted from human epidermal growth factor [68]. In this study, we generated different M1 variants based on the effect of histidine on VHH dissociation rate. **S5 Fig** shows the mTfR ELISA on M1 variants: M1_P96H_, M1_WT_ and M1_R100dH_. Comparing M1 variant binding to mTfR under normal physiological pH (pH = 7.2) and acidic pH (pH = 5.5), lower pH did not alter the dissociation of M1_WT_ to mTfR but substantially impacted the dissociation of M1_P96H_ to mTfR.

**Fig 2:**
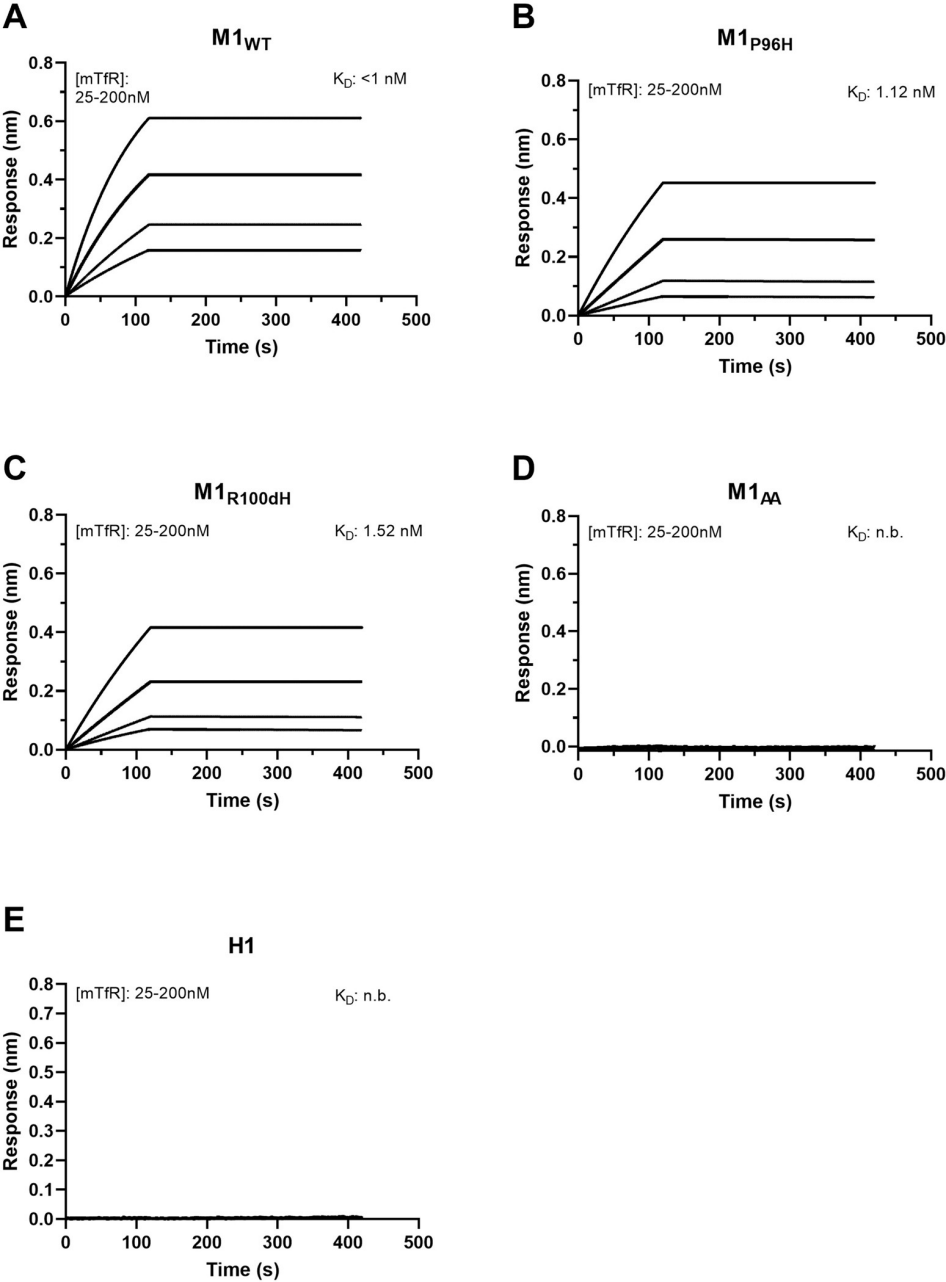
Affinity binding curves of the five M1-NT variants. M1_WT_-NT (**A**), M1_P96H_-NT (**B**), M1_R100dH_-NT (**C**), M1_AA_-NT (**D**) and H1-NT (**E**) binding affinity (K_D_) to mTfR. Using biolayer interferometry on an Octet Red96 system, association and dissociation rates were determined by immobilizing biotinylated VHH onto streptavidin-coated optical sensors. The M1-NT variant association and dissociation curves to mTfR were plotted and the affinity of each to mTfR was calculated. (n.b. = no binding).

Then the affinity of M1 variants to mTfR was measured using Octet. Based on Octet measurement, the affinity (KD) of M1_WT_-NT, M1_P96H_-NT, and M1_R100dH_-NT to mTfR were <1nM, 1.12nM, 1.52nM. While the affinity (KD) of M1_AA_-NT and H1-NT to mTfR was not detectable. **Fig 2** shows the association and dissociation curves of the M1_WT_-NT, M1_P96H_-NT, M1_AA_-NT, M1_R100dH_-NT and H1-NT to mTfR. M1_WT_-NT, M1_P96H_-NT, and M1_R100dH_-NT bond to mTfR well while M1_AA_-NT and H1-NT showed no binding. M1_WT_-NT and M1_P96H_-NT showed no binding to (Cys89-Phe760) human TfR extracellular domain (11020-H01H, SinoBiological) (**S6 Fig**).

### M1 variant screening for BBB transcytosis using neurotensin fusion and hypothermia assessment

**Fig 3:**
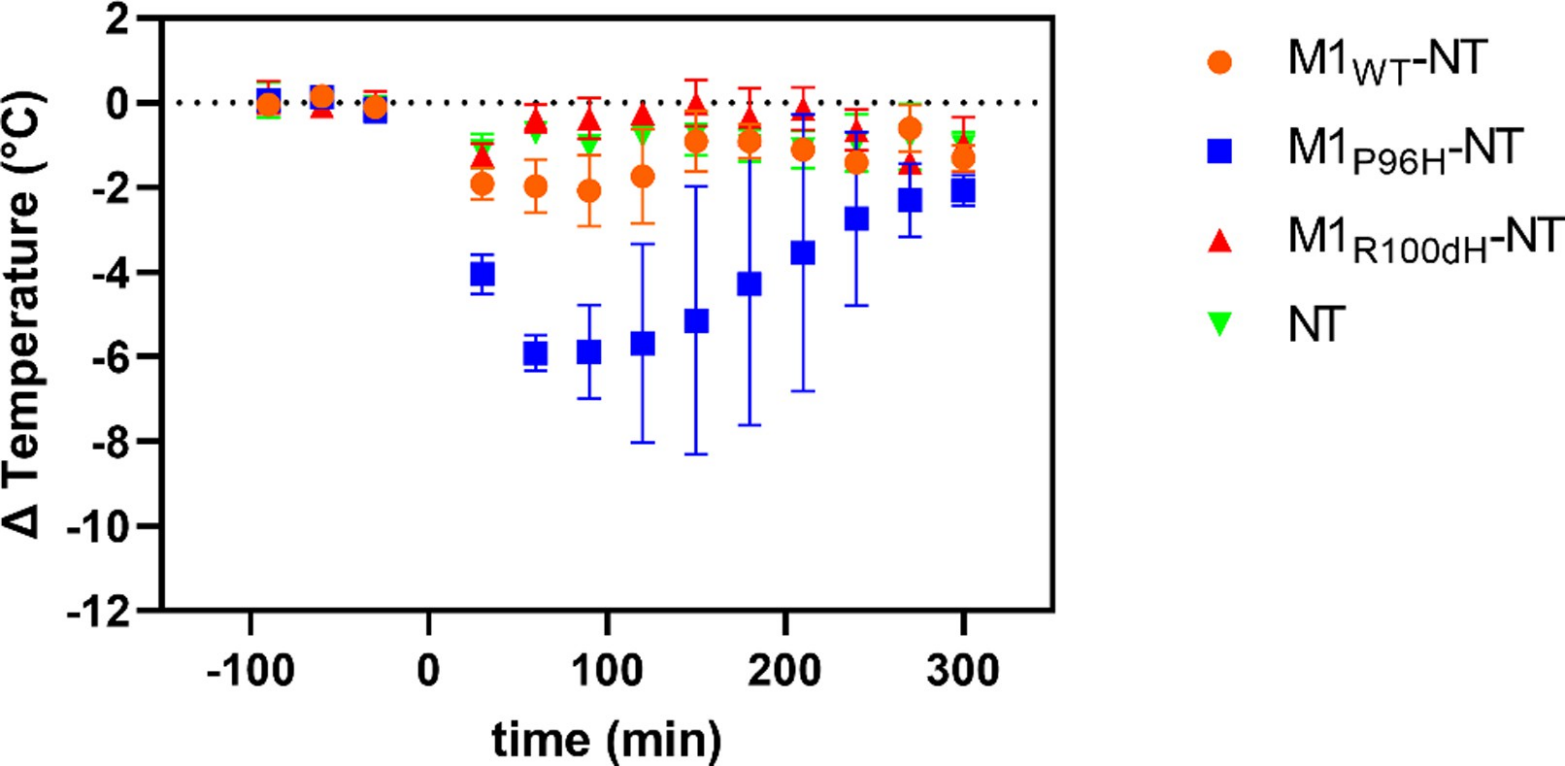
The use of NT fusion to VHHs for VHH screening via hypothermia as an indication of CNS target engagement. The M1 variants with different affinities to mTfR have different hypothermic effects. Among the three M1 variants injected to mice at the same dose 600nmol/kg body weight (n = 3 per group), M1_P96H_-NT produced the most prominent hypothermia effect, with a maximum temperature drop of about 6C° and duration for about 4hrs.

To assess BBB transcytosis ability, several M1 variants were fused to NT and screened based on the extent of hypothermia effects. Three M1 variant NT fusions, including M1_WT_-NT, M1_R100dH_-NT and M1_P96H_-NT were injected to WT mice. NT alone was injected into WT mice at the same molarity as an initial negative control. At a dose of 600nmol/kg body weight, the M1 variants with different binding affinities to mTfR show different hypothermia effects (**Fig 3**). M1_WT_-NT reduced temperatures by approximately 2C°, with an effect that lasted approximately 2 hours. M1_P96H_-NT, at the same dose, decreased temperature by 6C° with effects that lasted more than 4 hours, indicating that M1_P96H_-NT appeared to improve CNS penetration. NT alone did not show any hypothermia effect, indicating that peripherally administered NT alone does not appear to cross the BBB into the CNS. M1_R100dH_-NT gave less than 2C° of hypothermia effect. Mouse body temperatures were stable at baseline.

**Fig 4:**
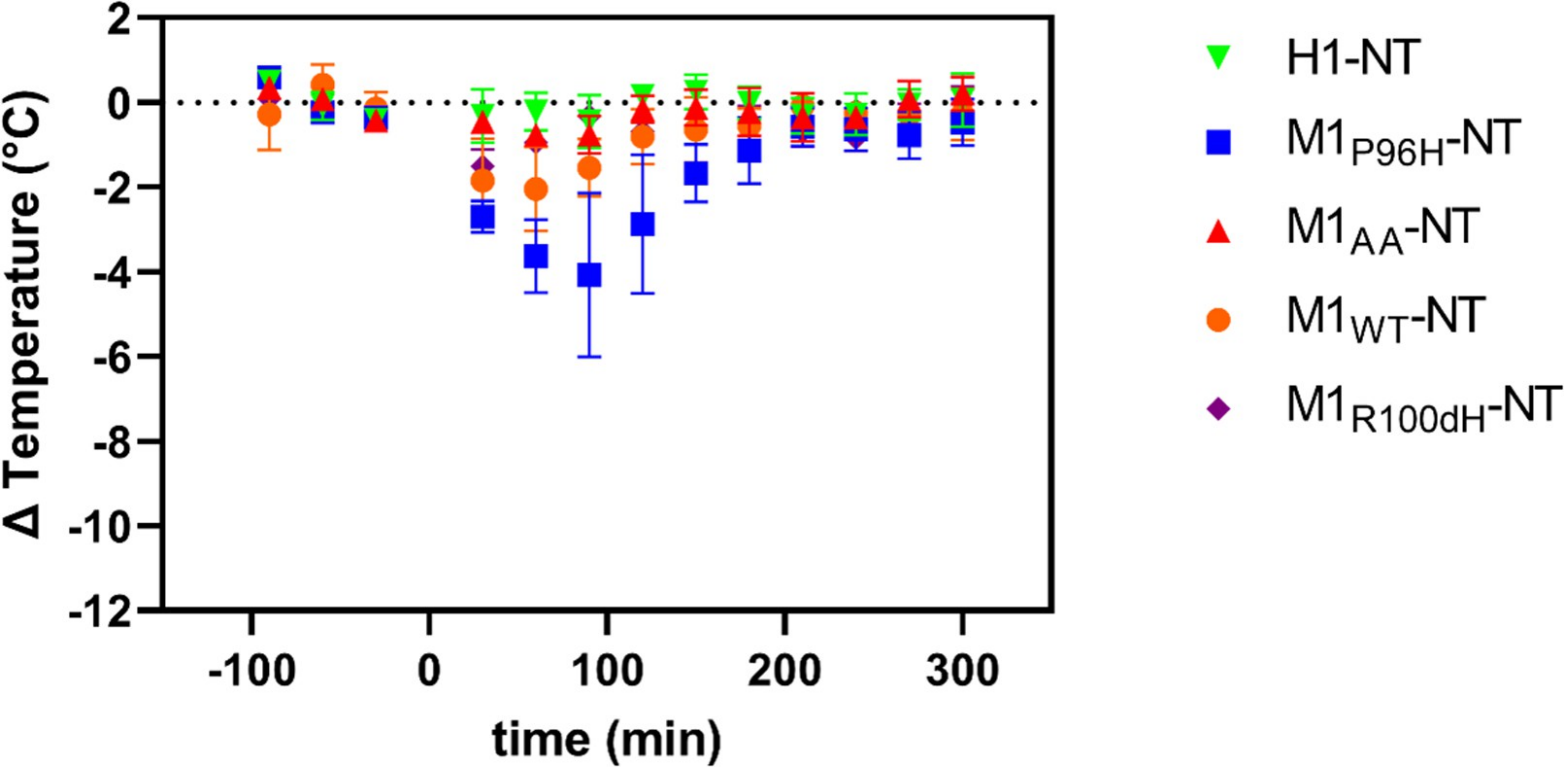
Hypothermic effect of M1 VHH-NT fusions in a randomized, blinded cohort. Five VHH-NT fusions M1_WT_-NT, M1_P96H_-NT, M1_AA_-NT, M1_R100dH_-NT and H1-NT were blinded by an independent investigator. Each blinded VHH-NT fusion was injected in a randomized fashion at a dose of 600nmol/kg body weight (n = 3 per group) and body temperature measured over the indicated time interval. Following complete data collection of the temperature measurements, the results were unblinded. Consistent with previous finding, M1_P96H_-NT gave the most prominent hypothermia effect.

To confirm and extend these findings, a blinded experiment was performed. The previous three M1 variants, plus a fourth mutant M1_AA_-NT that has minimal TfR binding and a different VHH that binds to human TfR called H1 were tested by an investigator blinded to the identity of the injected materials. M1_AA_-NT and H1-NT were tested at a higher molarity in a previous experiment and showed no obvious hypothermia effects (**S7 Fig**). The blinded experiment results were consistent with the previous finding: M1_P96H_-NT clearly reduced the temperature the most, with a maximum drop of about 4C°. The other M1-NTs and H1-NT did not reduce the temperature substantially (**Fig 4**). These results indicate that temperature measurement experiments facilitated the identification of an anti-mTfR VHH variant which induced substantial CNS effects.

### M1_P96H_-NT dose effect

**Fig 5:**
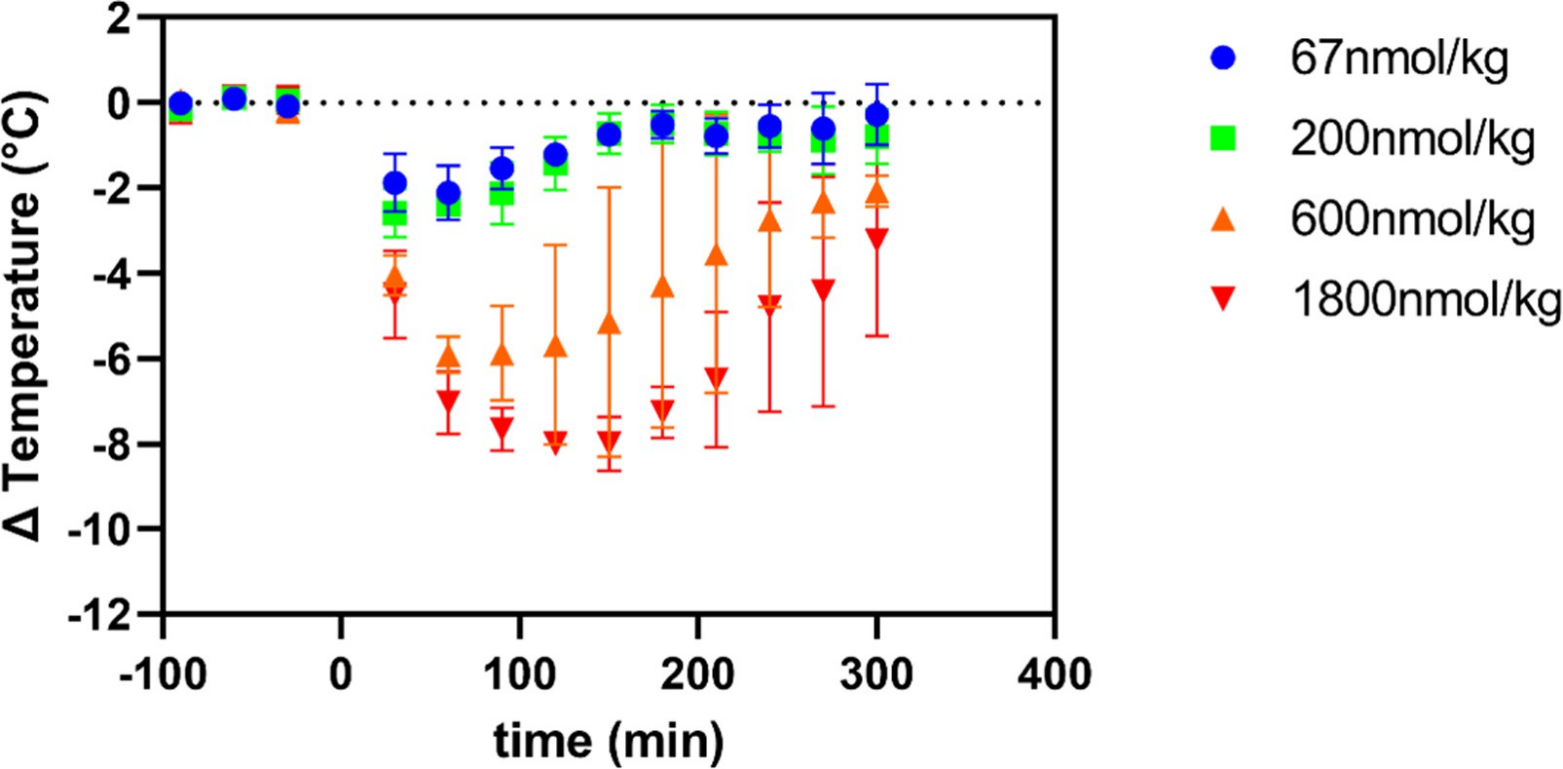
M1_P96H_-NT dose effect in WT mice after IV injection. This figure shows the dose effect of M1_P96H_-NT in WT mice after tail vein injection (n = 3 per group). The hypothermia effect was stronger and lasted longer time with dose increased from 67nmol/kg body weight to 1800nmol/kg body weight.

To understand whether the amount of M1_P96H_-NT injected to mice affects the extent of hypothermia effects, we injected M1_P96H_-NT to WT mice at four different doses: 67nmol/kg body weight, 200nmol/kg body weight, 600nmol/kg body weight, and 1800nmol/kg body weight. Twelve mice were used in this experiment, with three mice at each dose (**Fig 5**). At the two lower doses, there was minimal hypothermia effects. With increased M1_P96H_-NT concentration, the maximum temperature drop increased to about 8C° and also the duration of hypothermia was extended to as long as 5 hours. Thus, these was an apparently monotonic dose-response relationship between intravenous M1_P96H_-NT and hypothermia.

### Temperature effect of M1_P96H_-Triplet-NT and H1-Triplet-NT

**Fig 6:**
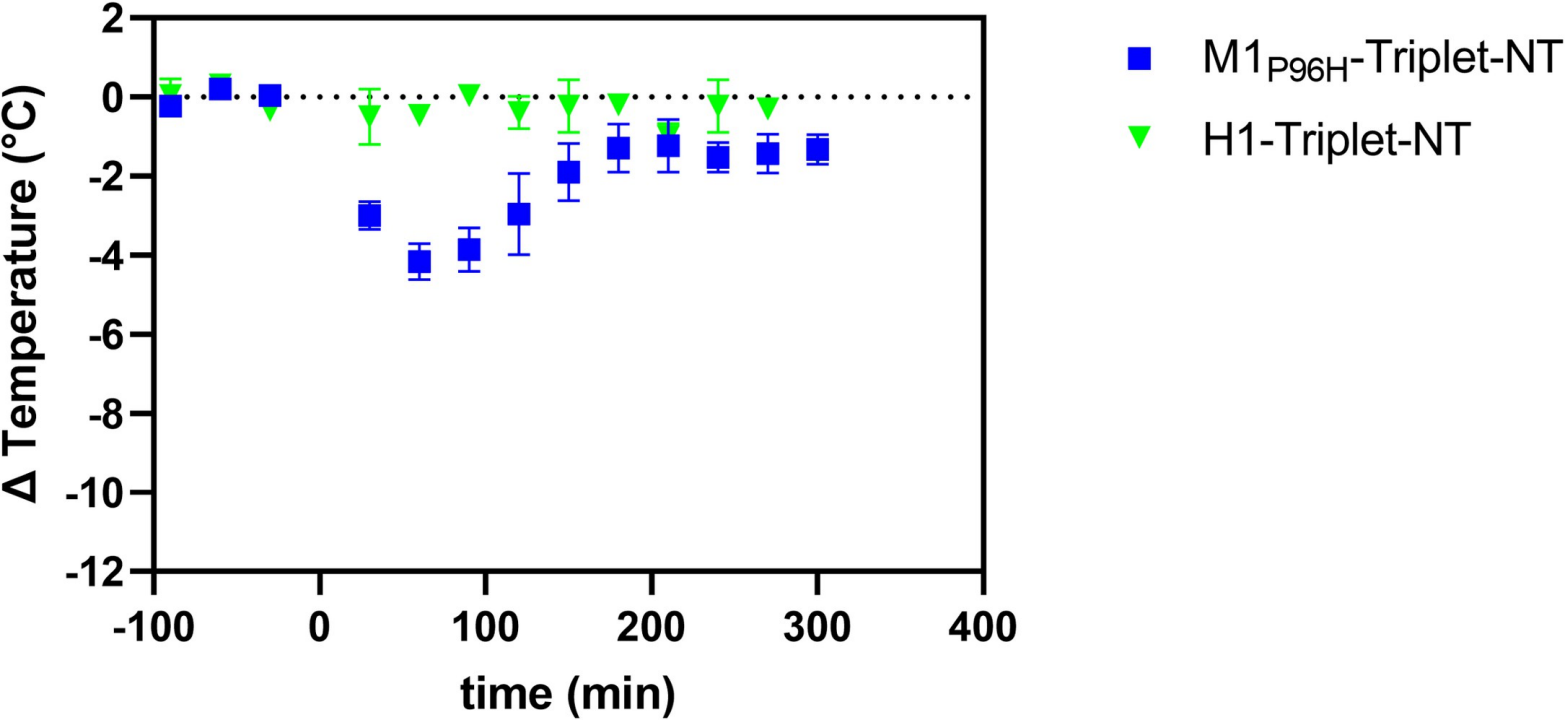
Measurement of M1_P96H_-Triplet-NT and H1-Triplet-NT hypothermia effect. M1_P96H_-Triplet-NT and H1-Triplet-NT were injected at dose of 600nmol/kg body weight (n = 3). M1_P96H_-Triplet-NT caused a temperature drop of about 4C° and H1-Triplet-NT caused no temperature drop.

Based on the octet results and temperature measurement, M1_P96H_ was chosen as the variant to use to assess ability to cross the BBB after conjugating to a payload. As a proof of concept, we used the anti-Aβ VHH dimer Nb3-Nb3 as the payload fused in a single polypeptide with M1_P96H_ and NT and named M1_P96H_-Triplet-NT. The Nb3-Nb3 dimer was also fused to H1 and NT and named H1-Triplet-NT as a negative control. The M1_P96H_-Triplet-NT and H1-Triplet-NT were injected intravenously into six WT mice (three mice for each Triplet-NT fusion). Body temperature was measured as described in the Temperature measurement section. **Fig 6** shows the temperature change after tail vein injection of M1_P96H_-Triplet-NT and H1-Triplet-NT. For M1_P96H_-Triplet-NT, there was about 4C° temperature drop after the intravenous injection and the hypothermia effect peaked at about 1 hour post injection. There was no substantial hypothermia effect after intravenous injection of H1-Triplet-NT. This result confirmed that after fusing to an anti-Aβ VHH dimer payload, the M1_P96H_-Triplet-NT was still able to get across the BBB and exert a CNS effect.

### Affinity measurement of VHH-Triplet

To confirm that fusion with Nb3-Nb3 did not affect the binding ability of M1_P96H_ and Nb3-Nb3 dimer to their targets, the affinity of M1_P96H_-triplet to mTfR and to Aβ were measured to be 1.63nM and 9.98nM using Octet (**S8 Fig**). The affinity of the M1_P96H_-triplet to mTfR was on the same order before and after the fusion. The affinity of H1-Triplet-647 to mTfR and Aβ was also assessed using Octet and measured to be no binding to mTfR and 9.44nM to Aβ (**S8 Fig**). It was verified that the fusion of M1_P96H_ and H1 to VHH dimer Nb3-Nb3 did not change their binding affinities.

To ensure that the dye labeling did not affect M1_P96H_-triplet binding to mTfR, a mTfR ELISA on M1_P96H_-triplet before and after Alexa 647 dye labelling was performed. The binding curves before and after labeling overlapped with each other (**S9 Fig**), indicating that the Alexa 647 dye labeling did not affect the binding to mTfR.

### Direct assessment of brain target engagement after intravenous injection of VHHs using confocal microscopy

VHH-Triplets were injected to APP/PS1 positive transgenic mice to directly assess brain target (Aβ) engagement through confocal microscopy. Ten transgenic mice were randomized into two groups, with five mice in each group. Five mice were injected with M1_P96H_-Triplet-647 and five mice were injected with H1-Triplet-647 as a negative control (H1-Triplet-647 does not bind to mTfR: **S8 Fig**).

**Fig 7:**
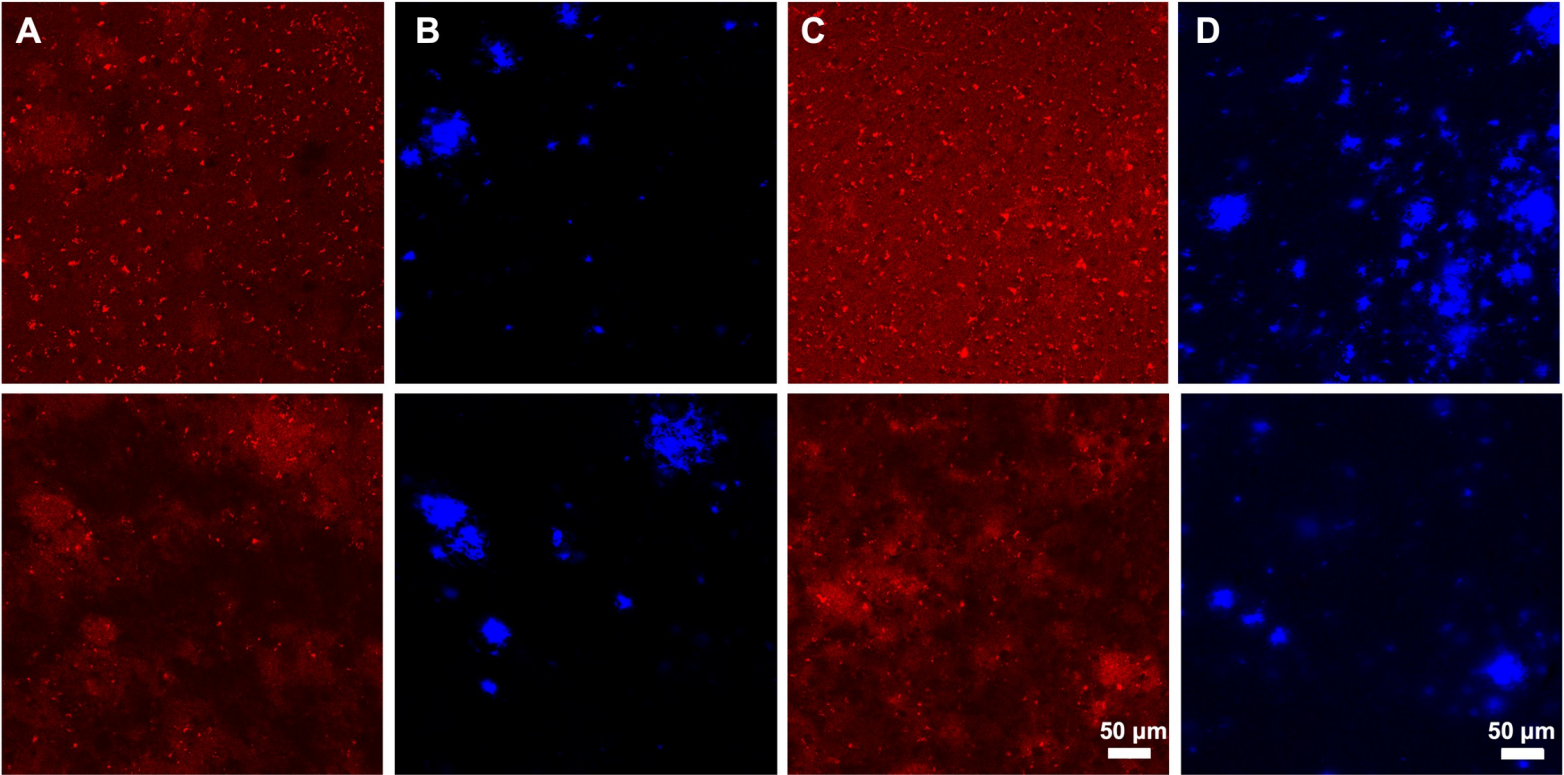
Representative immunofluorescent confocal microscopy of mice brains 2 hours after intravenous injection of Triplet-647. Mice brain sections were stained with X34 which labels the amyloid plaques. Representative 647 channel (**A**) and X34 channel (**B**) microscopy images of mice brains injected with H1-Triplet-647. Representative 647 channel (**C**) and X34 channel (**D**) microscopy images of mice injected with M1_P96H_-Triplet-647.

**Fig 8:**
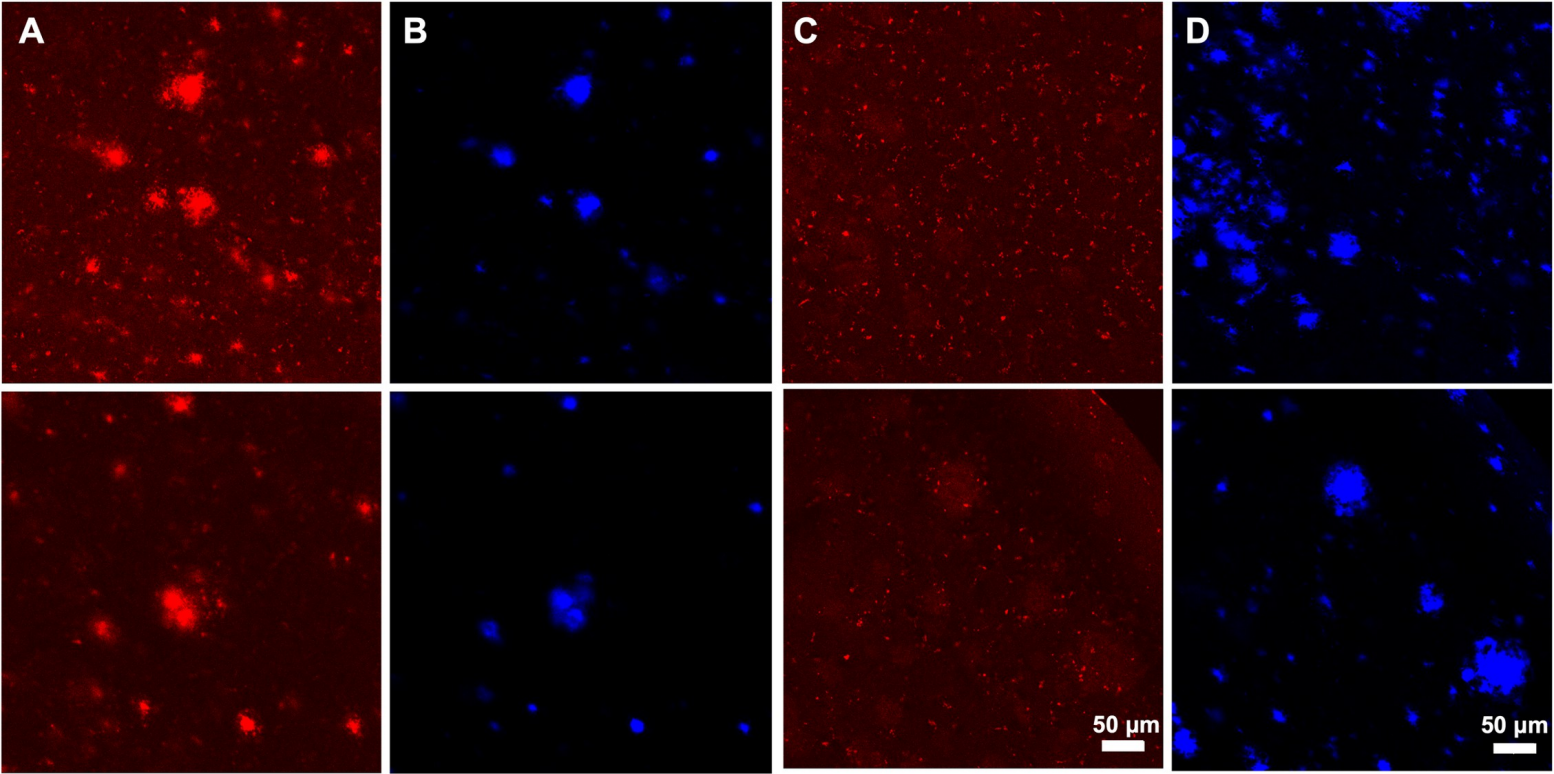
Representative immunofluorescent confocal microscopy of ex vivo APP/PS1 positive mouse brain sections. Mice brain sections were stained with X34 and M1_P96H_-Triplet-647 to label the amyloid plaques. Representative 647 channel (**A**) and X34 channel (**B**) microscopy images of the ex vivo brain sections labeled with M1_P96H_-Triplet-647. Representative 647 channel (**C**) and X34 channel (**D**) microscopy images of the ex vivo brain sections without M1_P96H_-Triplet-647 labeling.

After IV injection, the brain sections were stained with X34 to reveal the location of amyloid plaques (**Figs 7B and 7D and 8B and 8D)**. Because the NT-induced hypothermia effect was most prominent in the first two hours post injection in previous experiments using NT fusions, transgenic mice were sacrificed two hours post IV injection. Of note, when the VHH-Triplets were fused to NT and labeled with Alexa 647, they did not cause substantial temperature drop (**S10 Fig**), likely because the Alexa 647 labeling blocked the binding of NT to its receptor due to the presence of a lysine residue in the NT peptide. **Fig 7** shows exemplar confocal images of brains injected with M1_P96H_-triplet-647 (**Fig 7A and 7B**) or H1-triplet-647 (**Fig 7C and 7D**). From the exemplar images, it appeared that most of the 647 signals were similar to background, with some areas that had morphology consistent with amyloid plaque labeling (stained with X34, **Fig 7B and 7D**). However, surprisingly, the confocal imaging patterns were similar in both M1_P96H_-triplet-647 and H1-triplet-647 injected mice, suggesting a modest amount of non-specific BBB crossing and Aβ plaque binding plus background autofluorescence, rather than the expected extensive mTfR-mediated transcytosis and Aβ plaque binding.

To confirm that the lack of anti-Aβ plaque binding after IV injection by M1_P96H_-triplet-647 was not caused by the inability of the Nb3-Nb3 to label Aβ, an *ex vivo* labeling experiment was performed. We labeled brain sections from a naïve APP/PS1 positive mouse with M1_P96H_-Triplet-647 using modest concentrations and no antigen retrieval. The same brain sections were also stained with X34 to visualize the location of amyloid plaques. **Fig 8** shows substantially enhanced fluorescence signals labeled by M1_P96H_-triplet-647 in areas of plaques, indicating that the Nb3-Nb3 dimer part of M1_P96H_-Triplet-647 was able to bind to amyloid plaques at concentrations as low as 0.22 nM (**Figs 8A and 8B and S11**). Thus, the modest plaque labeling after intravenous injection with M1_P96H_-triplet-647 is not likely to be attributed to lack of plaque binding affinity. Autofluorescence was relatively modest in the 647 channel in sections labeled with only X34 (**Fig 8C and 8D**).

Amyloid plaque distribution was not consistent across mice and across regions of brain. To avoid subconscious bias in selecting regions for imaging and to assess for more subtle quantitative differences between M1_P96H_-triplet-647 and H1-triplet-647 plaque labeling, we performed randomized, blinded, automated analyses of the brain sections to objectively compare the confocal imaging results. Eleven or twelve confocal image stacks were acquired at randomly selected x, y coordinates in cortex (**S1 Fig**). Confocal images of brain sections were processed using ImageJ for quantitative assessment.

**Fig 9:**
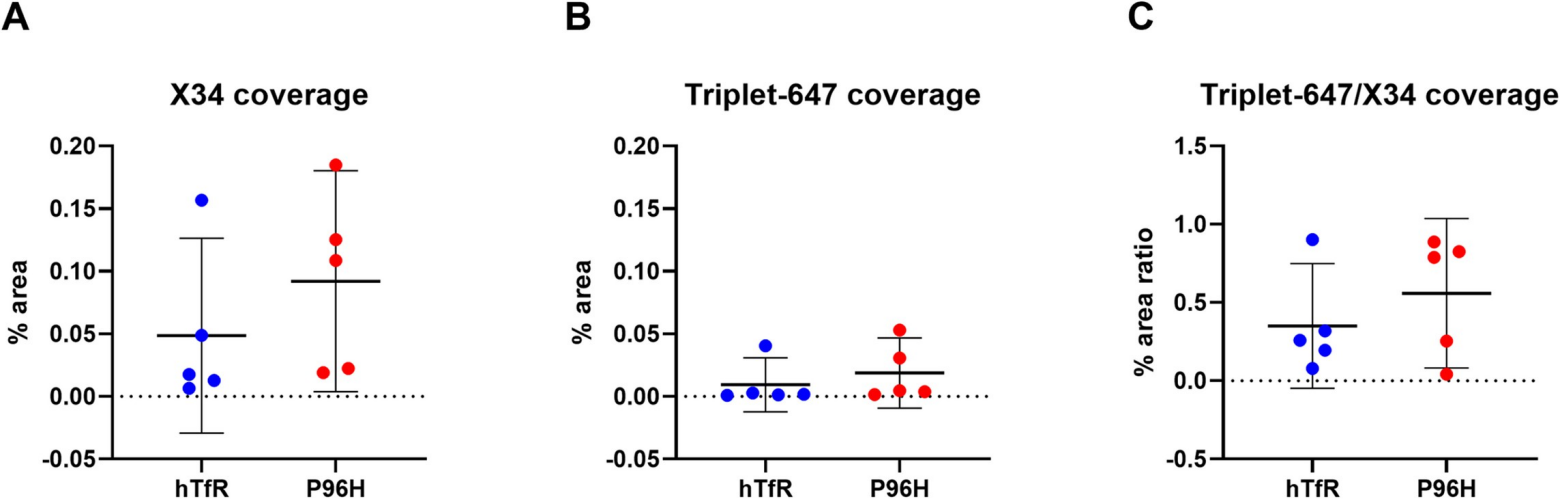
% area and % area ratio of X34 coverage and 647 coverage from mice injected with H1-triplet-647 or M1_P96H_-triplet-647. (**A**) The % area of X34 coverage in the X34 channel images. There was no significant difference for mice injected with H1-triplet-647 or M1_P96H_-triplet-647; unpaired student t-test, p = 0.3341 (**B**) The % area of Triplet-647 coverage in 647 channel images. There was no significant difference for mice injected with H1-triplet-647 or M1_P96H_-triplet-647; unpaired student t-test, p = 0.4815. (**C**) The % area ratio of Triplet-647/X34 coverage. There was no significant difference for mice injected with H1-triplet-647 or M1_P96H_-triplet-647; unpaired student t-test, p = 0.3788.

As described in **Materials and Methods**, the % area of X34 coverage, % area of Triplet-647 coverage and the % area ratio of Triplet-647/X34 coverage were averaged across the eleven to twelve images stacks acquired for each mouse brain. The average values of % area of each mouse brain was plotted in **Fig 9** Comparing mouse brains injected with M1_P96H_-triplet-647 or H1-triplet-647, the average values of % area of Triplet-647 coverage/% area X34 coverage had no significant difference (**Fig 9C**). There was also no significant difference for % area X34 coverage or % area Triplet-647 coverage (**Fig 9A and 9B**).

### Alternative confocal image processing–thresholding and intensity measurement

**Fig 10:**
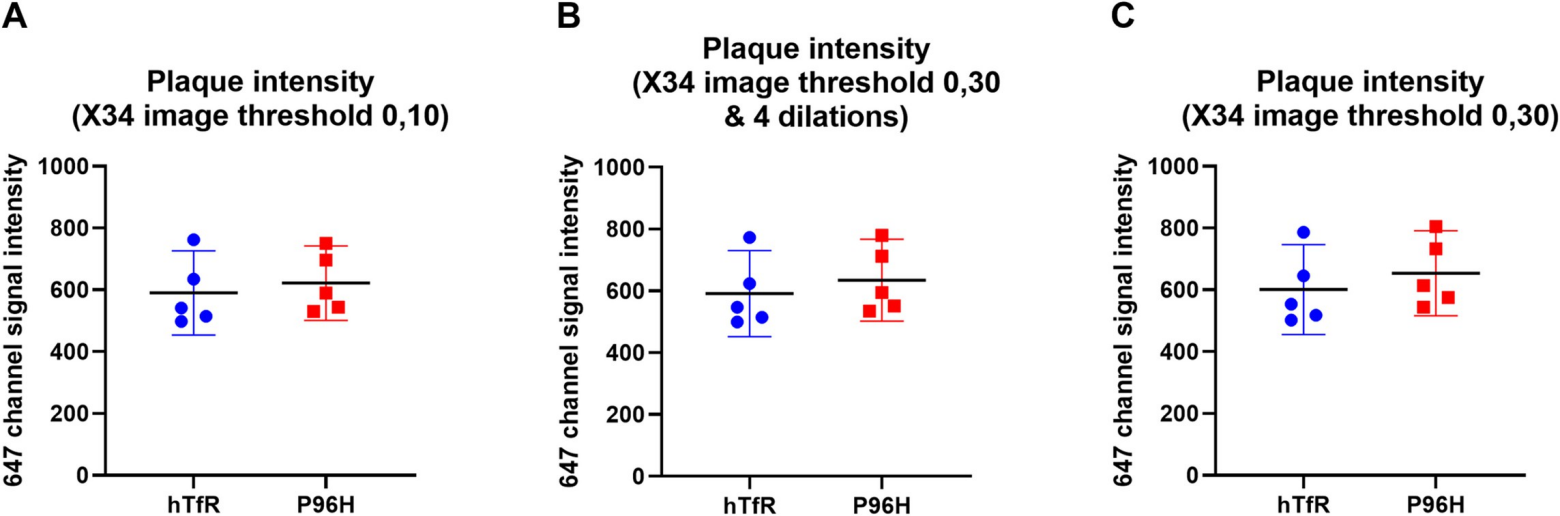
Averaged mean fluorescence in the 647 channel of plaques containing regions. (**A**) The threshold for X34 images was set to be at (0, 10) There was no significant difference between the mean signals from mice injected with H1-triplet-647 and mice injected with M1_P96H_-triplet-647; unpaired student t-test, p = 0.6404. (**B**) The threshold for X34 images was set to be at (0, 30), then the image was eroded for four times. There was no significant difference between the mean signals from mice injected with H1-triplet-647 and mice injected with M1_P96H_-triplet-647; unpaired student t-test, p = 0.5527. (**C**) The threshold for X34 images was set to be at (0, 30). There was no significant difference between the mean signals from mice injected with H1-triplet-647 and mice injected with M1_P96H_-triplet-647; unpaired student t-test, p = 0.4829.

To ensure the finding was not affected by the specific analysis method, confocal images of brain sections were analyzed in several alternative ways using ImageJ (**S12 Fig**). First, X34 images were alternatively thresholded at (0, 10) and (0, 30) and were converted to binary images. Because amyloid-beta pathology can extend beyond the boundaries of X34 positive fibrillar plaque cores, the X34 images thresholded at (0, 30) were also processed with dilation in ImageJ to add pixels from the edges of plaques. These plaque and peri-plaque regions identified on the X34 images were applied to the 647 channel images to calculate the mean 647 signal of these regions. The mean 647 signals were averaged across image stacks acquired for each mouse brain The average values of mean 647 signals of each brain were plotted in **Fig 10** There was no significant difference of the mean 647 signal between the two groups in any of these analyses. These findings confirmed that there was no detectible RMT of M1_P96H_-triplet-647 into the cortex, despite good evidence for a CNS effect for the same construct when fused to neurotensin.

### Alternative light microscopic detection

As a further alternative detection method, we used light microscopic immunohistochemistry with an anti-alpaca VHH secondary antibody on brain sections from mice injected with H1-triplet-647 or M1_P96H_-triplet-647 (**S1 File**). We found no evidence of plaque labeling using light microscopic immunohistochemistry either (**S13 Fig**).

### Interaction with transferrin

In the blood, transferrin is present in high concentrations. To test the hypothesis that the mTfR VHH M1 constructs interacted with transferrin itself during transferrin binding, we tested the affinity of M1 for the extracellular domain of mTfR in the presence or absence of mouse iron bound (holo) transferrin. There was no effect of 10 micrograms per ml of mouse holo transferrin on the affinity of M1 for mTfR (**S14 Fig**). This indicates that M1 likely binds to a different portion of the mTfR extracellular domain than transferrin and makes it unlikely that transferrin in the blood interferes with the binding of M1 constructs to mTfR at the BBB.

## Discussion

In this study, a modular system was designed to efficiently test the ability of anti-mTfR VHHs to cross the BBB using NT-induced hypothermia as a readout. Because only NT in the CNS can induce hypothermia effects [44, 47, 54, 69], experimentally measured hypothermia was used to infer the VHHs’ ability to cross the BBB through TfR-mediated transcytosis followed by VHH-NT fusion binding to neurotensin receptors. Using this NT-based screening system, this study successfully identified an anti-mTfR VHH variant, M1_P96H_, which has good binding properties to the mouse TfR and appeared to mediate BBB transport efficiently. To attempt to validate the ability of this anti-mTfR VHH to carry cargos across BBB, this anti-mTfR VHH was fused with an anti-Aβ VHH dimer and fused to NT. The H1 VHH was fused with anti-Aβ VHH dimer and NT as negative control. The M1_P96H_-Triplet-NT retained substantial hypothermia effects while the H1-Triplet-NT showed no hypothermia effect after intravenous injection. This finding confirmed the BBB transcytosis ability of M1_P96H_-Triplet-NT. However, the modular system for VHH screening does not appear to translate effectively into target binding in the cortex. To assess brain target engagement, M1_P96H_-triplet was conjugated to the fluorescent Alexa 647 dye and the dye conjugates were injected intravenously into APP/PS1 positive transgenic mice. A similar VHH triplet dye conjugate, H1-triplet which does not binding mTfR was used as a control. Surprisingly, there was no significant difference of the amyloid plaque binding between these two VHH triplet dye conjugates. This result was not likely due to failure of plaque binding, because the VHH triplet dye conjugates bound plaques well in *ex vivo* experiments. One possible explanation of our findings was that the VHH triplet dye conjugates did not effectively cross the BBB in the cortex; the hypothermia effect induced by NT might not correspond to target engagement in cortex.

The lack of correspondence between NT induced hypothermia after intravenous injection and brain target engagement could also be possibly explained by the concept that the BBB is not a unitary phenomenon, and the permeability of BBB may be different from brain region to brain region [70, 71]. The hypothalamus is the center for thermoregulation, and likely to be the site of action of NT in the brain. The preoptic area of hypothalamus contains microcircuitry through which cutaneous and core thermal signals are integrated for thermoregulation and temperature homeostasis [72, 73]. Studies have found that the interface between the median eminence and the arcuate nucleus of the hypothalamus is somewhat leaky to molecules in the circulation [74, 75]. Cheunsuang et al. confirmed the specialized nature of median eminence and medial arcuate nucleus BBB by testing the distribution of hydroxystilbamidine and wheat germ agglutinin after intravenous injection [74]. They found that hydroxystilbamidine was taken into the median eminence and medial part of the arcuate nucleus, while the wheat germ agglutinin diffusely distributed in the arcuate nucleus and median eminence following intravenous injection. Furthermore, Morita and Miyata reported the accessibility of low molecular weight blood-derived molecules to the parenchyma in the median eminence and arcuate nucleus [76]. The hypothermia effect caused by NT binding to NT receptor is likely to be mediated in the hypothalamus [54]. Young et al. explored the distribution of NT receptors in rat brains and found moderate to high density of NT receptors in hypothalamus. The hypothermia effect was induced when NT was injected to medial, lateral preoptic and anterior area of the hypothalamus. Thus, the dissociation between robust NT-mediated hypothermia effects vs. negligible binding to amyloid plaques in cortex after IV injection could be explained by a relatively leaky hypothalamic BBB: one hypothetical explanation for our results could be that the NT-induced hypothermia effect results from a combination of more permeable BBB and M1_P96H_ enhanced TfR-mediated transcytosis, while with only M1_P96H_ enhanced transcytosis there is not enough VHHs to label the amyloid plaques. Future studies will be required to directly test this possibility.

Additionally, in this study, the minimum required dose for NT induced temperature effect was about 20-fold higher than Stocki et al. [47]. The relationship between the amount of VHHs needed for NT-induced hypothermia CNS effect and the amount of VHHs required to label and visualize the amyloid plaques is unclear. It is possible that NT-induced hypothermia effect requires smaller amount of VHHs than visualizing amyloid plaque does. The absolute amount of anti-TfR positive controls and VHH-Triplet in the brain parenchyma after crossing the BBB was not measured directly in the study. To better understand the cause of lack of correspondence between NT induced hypothermia and Aβ binding, the absolute amount of VHHs in the brain parenchyma will need to be assessed using ELISA after separating brain capillaries and brain parenchyma [47, 77]. Meanwhile, the affinity of the anti-Aβ VHH Nb3-Nb3 was measured to be around 9-10nM, which is about 10-fold lower than the affinity of anti-TfR VHH M1_P96H_. Higher affinity Aβ binding antibodies would be required to assess the role of differential affinity for Aβ vs TfR. Because TfR is also expressed on other brain cells such as choroid plexus epithelial cells and neurons [78], it is possible that the M1_P96H_-Triplet-NT crosses the blood CSF barrier and engages with periventricular hypothalamic NT receptors more effectively than it cross the blood brain barrier to interact with parenchymal Aβ. Anti-TfR antibodies such as OX-26 and 8D3 could be tested as a positive control to explore the correlation between NT induced hypothermia and brain target (Aβ) engagement in the future [21, 23]. Additionally, although the Alexa 647 fluorescence staining could label *ex vivo* brain sections at sub nM concentration (**S11 Fig**), it might not be sensitive enough to detect the VHHs *in vivo*. Alternative VHH-triplet detection methods were explored by labeling the VHHs with an anti-alpaca VHH secondary antibody, followed by DAB staining (**S13 Fig**). However, this method did not improve the VHH detection sensitivity. Other staining methods will need to be explored for VHHs detection.

### Limitations

There are several limitations of this study. First, although the M1_P96H_-NT induced substantial hypothermia, the affinity and kinetics of this anti-mTfR VHH variant have not been fully optimized. Researchers have discovered anti-TfR antibodies with affinities ranging from sub nM to ~100nM which were able to cross the BBB through TfR-mediated transcytosis [19, 47, 79–81]. For example, Stocki, et al. reported a sub nM (0.63nM) binding affinity of their VNAR TXB2-hFc to TfR and reported that the VNAR was able to cross BBB and bind to brain targets [47]. Kariolis, et al. developed a TfR-binding Fc polypeptide with about 100nM affinity to human TfR and improved CNS transcytosis [81]. Niewoehner, et al. engineered a Fab fragment of an anti-mTfR antibody with affinity of around 20 nM and showed increased brain parenchyma delivery [79]. Boado, et al. assessed an anti-TfR monoclonal antibody 8D3 with affinity about 2.3nM [80]. This 8D3 antibody was shown to be able to cross the BBB [82]. Based on Yu, et al. [19], antibody affinity and dose need to be balanced for optimal RMT and BBB crossing. M1 variants with optimal affinity to TfR for BBB transcytosis will need to be explored. Also, **Fig 2** shows that the dissociation rate of the M1_P96H_ from mTfR was 1.33X10^-4^ which could be still too low. Based on Hultqvist et al., after the VHH binds to the TfR and the complex is internalized, the VHH needs to dissociate from the TfR to get released efficiently into the brain parenchyma [24]. When the dissociation rate is too slow, VHHs may not unbind from the TfR and not get released into the brain parenchyma. Second, we were not able to assess the hypothermia effect of M1_P96H_-Triplet-NT after dye labeling. This is because Alexa 647 dye was conjugated to M1_P96H_ -Triplet-NT through NHS ester reaction. 647 dye binds to the lysine group on NT and disables NT’s ability to induce hypothermia effect (**S10 Fig**). It is possible that dye modification with Alexa 647 affected BBB transcytosis even though it did not affect mTfR binding affinity *in vitro* (**S9 Fig**). Alternative labeling strategies will need to be tested in future experiments. Reduction in autofluorescence due to lipofuscin [83] may also improve the sensitivity of fluorescent dye-based detection methods. Third, to match the maximum hypothermia effect, 2hr post injection was chosen as the terminal time point to access brain target engagement. However, this time point may not be optimal. Multiple time points post injection will need to be tested to find the optimal terminal time point for brain target engagement studies. Fourth, the APP/PS1 mice used in the study can also develop astrogliosis [84]. It is possible that the astrogliosis around the plaques could impair BBB transcytosis. Fifth, we do not know the mTfR binding epitope of M1, though it does not appear to interfere with transferrin binding (**S14 Fig**). Also, it is formally possible, though unlikely, that older APP/PS1 transgenic mice have a less permeable BBB than young WT mice; we have not directly tested hypothermia effects in older APP/PS1 mice. A minor limitation is that the temperature measurement was conducted using an infrared thermometer. Although belly fur was removed to decrease variation and increase the accuracy and precision of temperature measurement, the use of infrared thermometer to measure belly temperature still introduces inter- and intra- user error. Also, the mice need to be anesthetized for acquisition of a steady temperature using the thermometer. Although we kept the anesthesia time short (about 20 sec) each time to minimize the influence of anesthesia on mouse body temperature. The use of anesthesia still introduced extra variations. Other temperature measurement methods such as the use of implanted thermometers which provide real-time temperature readings and eliminate interference with mice should be considered for future experiments. Finally, we acknowledge that only one brain section posterior to bregma was analyzed for each mouse. More brain sections for each mouse which cover additional brain areas, including hypothalamus, will need to be imaged and analyzed in the future.

### Future directions

There are many important future directions for this line of investigation. This manuscript showed that pH-dependent M1 variant P96H improves TfR-mediated BBB transcytosis. Utilizing this property, we are in the process of screening other pH-dependent VHH variants to improve anti-mTfR and anti hTfR-mediated BBB transcytosis.

Because the NT induced hypothermia effect does not give enough information about target engagement in brain areas like cortex, additional approaches to assess BBB transcytosis are needed. To allow high throughput testing and prediction of therapeutic and diagnostic agent delivery to the CNS, *in vitro* BBB models have been used. Cecchelli et al. developed a human *in vitro* BBB model using cord blood-derived hematopoietic stem cells [85]. This model shows good correlation between *in vitro* predicted ratio of unbound drug concentration in brain and *in vivo* ratio reported in humans. Shayan et al. made a murine *in vitro* BBB model using murine brain microvascular endothelial cells which also shows good correlation of compound permeability compared with *in vivo* values [86]. However, these *in vitro* models do not always reflect the *in vivo* BBB function. Garberg et al. evaluated multiple different *in vitro* models in comparison with an *in vivo* mouse brain uptake assay to understand the *in vitro* models’ potential to predict *in vivo* transport of compounds across BBB [49]. Low correlations between *in vitro* and *in vivo* data were obtained with a total of twenty-two compounds. Because of the complexity of the *in vivo* environment, *in vitro* BBB models were not recommended to be used in isolation to assess target engagement in brain [87]. There are many other methods to assess BBB transcytosis. For example, Stocki et al. tested the BBB transcytosis of a variable domain of new antigen receptors (VNAR) TXB2 by fractionating capillaries from the brain parenchyma and measured the concentration in capillaries and brain parenchyma [47]. Yu et al. assessed the uptake of an anti-TfR antibody by homogenizing the brain target areas and measuring the antibody concentration with ELISA [19]. Other screening platforms will be explored and tested in the future to facilitate of the discovery of optimal anti-mTfR VHHs.

Using RMT to transport payload across the BBB has limitations, such as downregulating the targeted receptors and competition with natural ligands which may affect the receptors’ normal physiological functions [79, 88, 89]. For example, Couch et al., found that anti-TfR antibodies could cause a reduction of reticulocyte count and acute clinical signs [88]. In this study, there were no apparent safety concerns raised during the experiments reported here. However, the safety profile of the VHHs will be formally assessed in the future to make sure that the binding of VHHs to the mTfR does not cause reticulocyte reduction or interfere with physiological iron uptake [90].

In this study, the VHHs were produced in *E*. *Coli*. and contaminated by endotoxins in the outer membrane of this gram-negative bacteria [64]. Extra steps were required to remove endotoxin from the VHHs. In the future, VHHs will be produced in yeast which has high yield for VHH production and avoid contaminating VHHs with endotoxins [91]. Also, the immunogenicity and toxicity profiles of these foreign protein constructs will be assessed.

The reduced size of VHHs does have some disadvantages compared to traditional monoclonal antibodies, such as shortening their blood half-life and reducing total brain exposure [27, 28]. Extension of VHH fusion protein half-life in the circulation may improve BBB transcytosis. The half-life of single VHHs and VHH triplets was measured to be 2–3 minutes in the mouse after IV injection [92]. The lack of differences in brain signal may be caused by the fast clearance of the VHHs, which did not allow enough VHHs to bind to the TfR on the brain endothelial cells. Conjugation of the VHHs to albumin binding domains, immunoglobulin Fc domains or PEG to slow down VHH clearance and enhance blood residence time will be tested in the future [93–95].

While not directly related to the main aims of the project involving development of brain MRI molecular contrast agents, the mechanisms underlying potential differences in BBB function in the hypothalamus vs. cortex are worthy of further investigation. To begin, target engagement of the Triplet-647 in hypothalamus will need to be assessed. The hypothalamus area of the brain sections will be imaged with confocal microscope and analyzed using ImageJ. Then, the M1_P96H_ could be conjugated to nanobodies against another widely distributed endogenous target such as the ATP-gated ion channel P2X7, which is widely distributed throughout the brain [96]. M1_P96H_-anti P2X7 VHH fusion would then be intravenously injected to mice and its target engagement in cortex and hypothalamus would be assessed and compared. Furthermore, different sizes of dextrans could be intravenously injected to the mice to understand the BBB penetration capacity in hypothalamus and other brain regions [76].

The ultimate goal of this study is to develop a family of molecular MRI contrast agents for diagnosis and assessment of neurodegenerative diseases. Misdiagnosis of neurodegenerative diseases is common because of their heterogeneous nature [97]. Early diagnosis of neurodegenerative diseases could help with early treatments and delay hospitalization, and accurate identification of target populations could help with the development of new treatments [97, 98]. In the future, we plan to generate anti-human TfR VHHs for diagnosis of neurodegenerative diseases in human patients. MRI has been widely used for imaging neurodegenerative diseases; however, structural MRI provides indirect and nonspecific measurements. Neurodegenerative diseases are characterized by abnormal accumulation of misfolded proteins including α-synuclein, tau, TDP-43 and Huntingtin in the CNS. Novel families of MRI contrast agents with VHH and IONPs would allow visualization of pathologically specific biomarkers in the living human brain: each VHH would bind to the misfolded proteins while the IONPs provide T1 MRI signals. We have characterized the *in vivo* pharmacokinetics of the contrast agent and optimized the MRI sequence for MR T1 imaging [92, 99], but have not optimized BBB transcytosis of contrast agents. IONPs have been widely in a research context as MRI molecular contrast agents but are not typically used in clinical practice. Liu et al., developed an oligomer-specific scFv antibody W20 conjugated superparamagnetic iron oxide nanoparticles which specifically bound to oligomers in transgenic mouse models of Parkinson’s disease or Huntington’s disease and provided MRI signals [100]. Sillerud et al. synthesized an anti-AβPP conjugated superparamagnetic iron oxide nanoparticle for MRI detection of amyloid plaques in AD [101]. However, none of these contrast agents have provided optimal BBB penetration to allow high quality *in vivo* imaging. The ability of molecular contrast agents to cross the BBB and bind to brain targets in sufficient quantity to give conspicuous MRI signals will need be tested with *in vivo* MR imaging in the future.

## Conclusions

This study used a NT based modular system to screen anti-mTfR VHHs for TfR-mediated transcytosis across BBB. A M1 variant, M1_P96H_, was identified with good performance in inducing hypothermia, an effect which requires crossing the BBB. This M1 variant was fused to the anti-Aβ VHH dimer and labeled with Alexa 647. Surprisingly, however, the dye labelled VHH did not show detectible labeling of amyloid plaques compared with controls after intravenous injection into transgenic mice. Reasons for the lack of correspondence between NT-induced hypothermia effect and Aβ visualization will need to be explored. Additional methods to assess VHH BBB transcytosis will need to be developed for screening VHHs to facilitate the development of MRI molecular contrast agents.

## Supporting information

S1 Fig Schematic graph for the manual unbiased random selection method for confocal image acquisition. This schematic graph shows the logic of picking coordinates for confocal images acquisition. The right hemisphere of the brain was equally divided into ten parts in both x and y directions. (x, y) coordinates were randomly generated using a random number generator. The microscope stage was moved to the target (x, y) coordinates using the stage rulings. When (x, y) coordinates fell into the areas of cortex, images were taken and cropped to include only cortical areas. When (x, y) coordinates fell out of the cortex the coordinates were discarded and no images were taken. Source of the mouse brain: biorender.com.
(TIF)

S2 Fig The hypothermia effect of M1_WT_-NT before and after endotoxin removal. Endotoxin removal from in M1_WT_-NT preparations from E. coli decreased hypothermia effects at time 30min, 1hr and 1.5hr after IV injection of the M1_WT_ -NT (n = 3 per group). The maximum hypothermic effect of 600nmol/kg body weight of M1_WT_ -NT was approximately 2C° less after endotoxin removal.
(TIF)

S3 Fig Post purification assessment of the five VHH-NT fusions M1_WT_-NT, M1_P96H_-NT, M1_AA_-NT, M1_R100dH_-NT and H1-NT. The purity of the monomer VHH by (a) SDS-PAGE gel and exemplar (b) size-exclusion chromatography over a Superdex75 column for M1WT-NT indicating >95% purity and homogeneity of the material.
(TIF)

S4 Fig Post purification assessment of M1_P96H_-triplet-NT. The purity of the triplet VHH by (a) SDS-PAGE gel and exemplar (b) size-exclusion chromatography over a Superdex75 column for M1_P96H_-Triplet-NT indicating >95% purity and homogeneity of the material.
(TIF)

S5 Fig Histidine mutations impart a pH dependent dissociation effect in M1 VHH variants. M1 variants (a) M1_WT_, (b) M1_P96H_ and (c) M1_R100dH_ were incubated on mTfR coated ELISA plates, followed by a stringent wash with 1x PBS buffer at pH 7.2 or pH 5.5. Following pH dependent washing, the bound VHH was detected with an anti-alpaca-peroxidase antibody and the reaction terminated by addition of 1M HCl. Error bars represent the standard deviation of the mean values at each data point.
(TIF)

S6 Fig mTfR and hTfR ELISA on M1WT-NT (a) and M1P96H-NT (b). VHHs were incubated with mTfR or hTfR absorbed to ELISA plates and detected using an anti-alpaca-peroxidase antibody to determine the impact of fluorophore conjugation. The curves show no measurable binding of M1WT-NT (a) and M1P96H- NT (b) to human TfR extracellular domain.
(TIF)

S7 Fig Lack of hypothermic effect with mTfR non-binding VHH-NT fusions. M1_AA_-NT and H1-NT were injected at doses of 1400nmol/kg body weight. M1_AA_-NT and H1-NT lack prominent hypothermia effects (n = 3 per group).
(TIF)

S8 Fig Affinity binding curves of M1_P96H_-triplet-NT and H1-triplet-NT binding affinity (KD) to mTfR and Aβ. Using biolayer interferometry on an Octet Red96 system, association and dissociation rates were determined by immobilizing biotinylated VHH (a, c) or biotinylated amyloid beta (b, d) onto streptavidin-coated optical sensors. M1_P96H_-triplet-NT association and dissociation curve to (a) mTfR and (b) Aβ. The affinity of M1_P96H_-triplet-NTwas measured to be 1.63nM to mTfR and 9.98nM to Aβ. H1-triplet-NT association and dissociation curve to (c) mTfR and (d) Aβ. The affinity of H1-triplet-NT was measured to be 9.44nM to Aβ and no binding to mTfR. (n.b. = no binding).
(TIF)

S9 Fig mTfR ELISA on M1P96H-Triplet before and after 647 labelling. VHH was incubated with mTfR absorbed to ELISA plates and detected using an anti-alpaca-peroxidase antibody to determine the impact of fluorophore conjugation. The near overlapping binding curves indicate a lack of effect following fluorophore labelling. Error bars represent the standard deviation of the mean values at each data point.
(TIF)

S10 Fig Measurement of M1_P96H_-Triplet-NT-647 hypothermia effect. M1_P96H_-Triplet-NT-647 was injected at dose of 600nmol/kg body weight (n = 3). M1_P96H_-Triplet-NT-647 did not cause temperature drop.
(TIF)

S11 Fig Representative immunofluorescent confocal microscopy of ex vivo APP/PS1 positive mouse brain sections. Mice brain sections were stained with M1_P96H_-Triplet-647 which labels the amyloid plaques. The representative 647 channel microscopy images of the ex vivo brain sections show substantial amyloid plaques labeling with M1_P96H_-Triplet-647 at concentration of 22.2nM, 2.22nM, and 0.22nM.
(TIF)

S12 Fig Quantitative analysis procedure for confocal images with thresholding followed by automated plaque selection and mean 647 channel signal calculation. An APP/PS1 positive naïve brain slice with ex vivo M1_P96H_-Triplet-647 labeling was used as the example. Column a. shows a representative confocal microscope image of cortex X34 staining. Column b. shows the X34 labeled images after thresholding. Column c. shows automated selection of regions of plaques based on the thresholded images. Column d. shows the application of selected areas (from thresholded X34 images) to the 647 channel images.
(TIF)

S13 Fig Light microscopic immunohistochemical assessment of mouse brains 2 hours after intravenous injection of triplets. Anti-alpaca VHH secondary antibody against triplet-647 was used for immunohistochemistry. Mouse brains injected with H1-triplet-647 (a) and M1_P96H_-triplet-647 (b). Scale bar is 200 μm.
(TIF)

S14 Fig ELISA on anti-mTfR VHH M1 in the presence or absence of 10ug/ml of iron-bound (holo) mouse transferrin. No change in binding affinity of mTfRnb-M1 to mTfR in the presence or absence of 10ug/ml of. Anti-mTfR VHH M1 was incubated with mTfR absorbed to ELISA plates and detected using an anti-alpaca-peroxidase antibody to determine the impact of mouse transferrin presence. The binding curves nearly overlap, indicating the presence or absence of mouse transferrin did not affect the binding affinity of M1 to mTfR. Error bars represent the standard deviation of the mean values at each data point.
(TIF)

S1 File Alternative light microscopic detection. Staining was performed to visualize amyloid plaques using the Anti-alpaca VHH secondary nanobody. Free-floating 50 μm tissue was rinsed with PBS for three times. Then, tissues were incubated in 2% normal goat serum (NGS) with 0.1% Triton X-100 in PBS for 60min to block non-specific binding. After blocking, tissue was rinsed with 1X PBS for three times then incubated in 0.5% NGS with 0.1% Triton X-100 in PBS with 0.8mg/ml Anti-alpaca VHH secondary antibody (Jackson ImmunoResearch, 128-065-232) for 60min. After antibody incubation, the tissue was rinsed with PBS for three times, followed by incubation with ABC Elite (Vector Laboratories) at a 1:400 dilution in PBS for 60min. The tissue was then rinsed with PBS for three times and developed with 3,3’-diaminobenzidine (#D5905; Sigma-Aldrich). The tissue was rinsed with 1X PBS for three times then mounted onto positively charged slides (EF15978Z, Daigger®). The mounted tissue sections were allowed to dry overnight at room temperature and dehydrated using ethanol series. Then, the sections were cover slipped using VectaMount® Permanent Mounting Medium (H-5000-60).
(PDF)

S1 Raw images Blot images of SDS-PAGE. (PDF)

## References

[1] LaFerla FM, Oddo S. Alzheimer’s disease: Abeta, tau and synaptic dysfunction. Trends Mol Med. 2005;11(4):170–6. doi: 10.1016/j.molmed.2005.02.009 . 15823755

[2] The Alzheimer’s Association. 2021 Alzheimer’s disease facts and figures. Alzheimers Dement. 2021;17(3):327–406. Epub 20210323. doi: 10.1002/alz.12328 . 33756057

[3] Graham WV, Bonito-Oliva A, Sakmar TP. Update on Alzheimer’s Disease Therapy and Prevention Strategies. Annu Rev Med. 2017;68:413–30. Epub 2017/01/19. doi: 10.1146/annurev-med-042915-103753 . 28099083

[4] Takizawa C, Thompson PL, van Walsem A, Faure C, Maier WC. Epidemiological and economic burden of Alzheimer’s disease: a systematic literature review of data across Europe and the United States of America. J Alzheimers Dis. 2015;43(4):1271–84. doi: 10.3233/JAD-141134 . 25159675

[5] Risacher SL, Saykin AJ. Neuroimaging and other biomarkers for Alzheimer’s disease: the changing landscape of early detection. Annu Rev Clin Psychol. 2013;9:621–48. Epub 20130107. doi: 10.1146/annurev-clinpsy-050212-185535 ; PubMed Central PMCID: PMC3955298. 23297785 PMC3955298

[6] Clark CM, Davatzikos C, Borthakur A, Newberg A, Leight S, Lee VM, et al. Biomarkers for early detection of Alzheimer pathology. Neurosignals. 2008;16(1):11–8. Epub 20071205. doi: 10.1159/000109754 ; PubMed Central PMCID: PMC2866153. 18097155 PMC2866153

[7] Beach TG, Monsell SE, Phillips LE, Kukull W. Accuracy of the clinical diagnosis of Alzheimer disease at National Institute on Aging Alzheimer Disease Centers, 2005–2010. J Neuropathol Exp Neurol. 2012;71(4):266–73. doi: 10.1097/NEN.0b013e31824b211b ; PubMed Central PMCID: PMC3331862. 22437338 PMC3331862

[8] Wurtman R. Biomarkers in the diagnosis and management of Alzheimer’s disease. Metabolism. 2015;64(3 Suppl 1):S47–50. Epub 20141030. doi: 10.1016/j.metabol.2014.10.034 . 25468144

[9] ten Kate M, Redolfi A, Peira E, Bos I, Vos SJ, Vandenberghe R, et al. MRI predictors of amyloid pathology: results from the EMIF-AD Multimodal Biomarker Discovery study. Alzheimer’s Research & Therapy. 2018;10(1):100. doi: 10.1186/s13195-018-0428-1 30261928 PMC6161396

[10] Morris E, Chalkidou A, Hammers A, Peacock J, Summers J, Keevil S. Diagnostic accuracy of 18F amyloid PET tracers for the diagnosis of Alzheimer’s disease: a systematic review and meta-analysis. European Journal of Nuclear Medicine and Molecular Imaging. 2016;43(2):374–85. doi: 10.1007/s00259-015-3228-x 26613792 PMC4700091

[11] Lee G, Dallas S, Hong M, Bendayan R. Drug transporters in the central nervous system: brain barriers and brain parenchyma considerations. Pharmacol Rev. 2001;53(4):569–96. . 11734619

[12] Pardridge WM. Drug and gene targeting to the brain with molecular Trojan horses. Nat Rev Drug Discov. 2002;1(2):131–9. doi: 10.1038/nrd725 . 12120094

[13] Oller-Salvia B, Sánchez-Navarro M, Giralt E, Teixidó M. Blood-brain barrier shuttle peptides: an emerging paradigm for brain delivery. Chem Soc Rev. 2016;45(17):4690–707. doi: 10.1039/c6cs00076b . 27188322

[14] Malakoutikhah M, Teixidó M, Giralt E. Shuttle-mediated drug delivery to the brain. Angew Chem Int Ed Engl. 2011;50(35):7998–8014. Epub 20110630. doi: 10.1002/anie.201006565 . 21721080

[15] Kinoshita M, McDannold N, Jolesz FA, Hynynen K. Targeted delivery of antibodies through the blood-brain barrier by MRI-guided focused ultrasound. Biochem Biophys Res Commun. 2006;340(4):1085–90. Epub 20051227. doi: 10.1016/j.bbrc.2005.12.112 . 16403441

[16] McCarty DM, DiRosario J, Gulaid K, Muenzer J, Fu H. Mannitol-facilitated CNS entry of rAAV2 vector significantly delayed the neurological disease progression in MPS IIIB mice. Gene Therapy. 2009;16(11):1340–52. doi: 10.1038/gt.2009.85 19587708 PMC4289609

[17] Bien-Ly N, Yu YJ, Bumbaca D, Elstrott J, Boswell CA, Zhang Y, et al. Transferrin receptor (TfR) trafficking determines brain uptake of TfR antibody affinity variants. J Exp Med. 2014;211(2):233–44. Epub 20140127. doi: 10.1084/jem.20131660 ; PubMed Central PMCID: PMC3920563. 24470444 PMC3920563

[18] Gupta Y, Jain A, Jain SK. Transferrin-conjugated solid lipid nanoparticles for enhanced delivery of quinine dihydrochloride to the brain. J Pharm Pharmacol. 2007;59(7):935–40. doi: 10.1211/jpp.59.7.0004 . 17637187

[19] Yu YJ, Zhang Y, Kenrick M, Hoyte K, Luk W, Lu Y, et al. Boosting brain uptake of a therapeutic antibody by reducing its affinity for a transcytosis target. Sci Transl Med. 2011;3(84):84ra44. doi: 10.1126/scitranslmed.3002230 . 21613623

[20] Wiley DT, Webster P, Gale A, Davis ME. Transcytosis and brain uptake of transferrin-containing nanoparticles by tuning avidity to transferrin receptor. Proc Natl Acad Sci U S A. 2013;110(21):8662–7. Epub 20130506. doi: 10.1073/pnas.1307152110 ; PubMed Central PMCID: PMC3666717. 23650374 PMC3666717

[21] Jefferies WA, Brandon MR, Hunt SV, Williams AF, Gatter KC, Mason DY. Transferrin receptor on endothelium of brain capillaries. Nature. 1984;312(5990):162–3. doi: 10.1038/312162a0 . 6095085

[22] Pardridge WM, Buciak JL, Friden PM. Selective transport of an anti-transferrin receptor antibody through the blood-brain barrier in vivo. J Pharmacol Exp Ther. 1991;259(1):66–70. . 1920136

[23] Kissel K, Hamm S, Schulz M, Vecchi A, Garlanda C, Engelhardt B. Immunohistochemical localization of the murine transferrin receptor (TfR) on blood–tissue barriers using a novel anti-TfR monoclonal antibody. Histochemistry and Cell Biology. 1998;110(1):63–72. doi: 10.1007/s004180050266 9681691

[24] Hultqvist G, Syvänen S, Fang XT, Lannfelt L, Sehlin D. Bivalent Brain Shuttle Increases Antibody Uptake by Monovalent Binding to the Transferrin Receptor. Theranostics. 2017;7(2):308–18. Epub 20170101. doi: 10.7150/thno.17155 ; PubMed Central PMCID: PMC5197066. 28042336 PMC5197066

[25] Song Y, Du D, Li L, Xu J, Dutta P, Lin Y. In Vitro Study of Receptor-Mediated Silica Nanoparticles Delivery across Blood-Brain Barrier. ACS Appl Mater Interfaces. 2017;9(24):20410–6. Epub 20170612. doi: 10.1021/acsami.7b03504 ; PubMed Central PMCID: PMC5533093. 28541655 PMC5533093

[26] Hamers-Casterman C, Atarhouch T, Muyldermans S, Robinson G, Hamers C, Songa EB, et al. Naturally occurring antibodies devoid of light chains. Nature. 1993;363(6428):446–8. doi: 10.1038/363446a0 . 8502296

[27] Hassanzadeh-Ghassabeh G, Devoogdt N, De Pauw P, Vincke C, Muyldermans S. Nanobodies and their potential applications. Nanomedicine (Lond). 2013;8(6):1013–26. Epub 2013/06/05. doi: 10.2217/nnm.13.86 . 23730699

[28] Harmsen MM, De Haard HJ. Properties, production, and applications of camelid single-domain antibody fragments. Appl Microbiol Biotechnol. 2007;77(1):13–22. Epub 2007/08/21. doi: 10.1007/s00253-007-1142-2 ; PubMed Central PMCID: PMC2039825. 17704915 PMC2039825

[29] van der Linden RH, Frenken LG, de Geus B, Harmsen MM, Ruuls RC, Stok W, et al. Comparison of physical chemical properties of llama VHH antibody fragments and mouse monoclonal antibodies. Biochim Biophys Acta. 1999;1431(1):37–46. doi: 10.1016/s0167-4838(99)00030-8 . 10209277

[30] Oliveira S, van Dongen GA, Stigter-van Walsum M, Roovers RC, Stam JC, Mali W, et al. Rapid visualization of human tumor xenografts through optical imaging with a near-infrared fluorescent anti-epidermal growth factor receptor nanobody. Mol Imaging. 2012;11(1):33–46. . 22418026

[31] Stijlemans B, Conrath K, Cortez-Retamozo V, Van Xong H, Wyns L, Senter P, et al. Efficient targeting of conserved cryptic epitopes of infectious agents by single domain antibodies. African trypanosomes as paradigm. J Biol Chem. 2004;279(2):1256–61. Epub 20031003. doi: 10.1074/jbc.M307341200 . 14527957

[32] Rossotti MA, Bélanger K, Henry KA, Tanha J. Immunogenicity and humanization of single-domain antibodies. Febs j. 2021. Epub 20210309. doi: 10.1111/febs.15809 . 33751827

[33] Ackaert C, Smiejkowska N, Xavier C, Sterckx YGJ, Denies S, Stijlemans B, et al. Immunogenicity Risk Profile of Nanobodies. Front Immunol. 2021;12:632687. Epub 2021/03/27. doi: 10.3389/fimmu.2021.632687 ; PubMed Central PMCID: PMC7985456. 33767701 PMC7985456

[34] Liu J, Hong H, Shi J, Xie Y, Lu Z, Liu Z, et al. Dinitrophenol-mediated modulation of an anti-PD-L1 VHH for Fc-dependent effector functions and prolonged serum half-life. Eur J Pharm Sci. 2021;165:105941. Epub 20210710. doi: 10.1016/j.ejps.2021.105941 . 34256102

[35] Liu Y, Huang H. Expression of single-domain antibody in different systems. Applied Microbiology and Biotechnology. 2018;102(2):539–51. doi: 10.1007/s00253-017-8644-3 29177623

[36] Food and Drug Administration. FDA approved caplacizumab-yhdp. February 2019.

[37] Ishiwatari-Ogata C, Kyuuma M, Ogata H, Yamakawa M, Iwata K, Ochi M, et al. Ozoralizumab, a Humanized Anti-TNFα NANOBODY® Compound, Exhibits Efficacy Not Only at the Onset of Arthritis in a Human TNF Transgenic Mouse but Also During Secondary Failure of Administration of an Anti-TNFα IgG. Frontiers in Immunology. 2022;13. doi: 10.3389/fimmu.2022.853008 35273620 PMC8902368

[38] D’Huyvetter M, Vos J, Caveliers V, Vaneycken I, Heemskerk J, Duhoux FP, et al. Phase I Trial of (131)I-GMIB-Anti-HER2-VHH1, a New Promising Candidate for HER2-Targeted Radionuclide Therapy in Breast Cancer Patients. J Nucl Med. 2021;62(8):1097–105. Epub 20201204. doi: 10.2967/jnumed.120.255679 . 33277400

[39] Sheff J, Wang P, Xu P, Arbour M, Masson L, van Faassen H, et al. Defining the epitope of a blood–brain barrier crossing single domain antibody specific for the type 1 insulin-like growth factor receptor. Scientific Reports. 2021;11(1):4284. doi: 10.1038/s41598-021-83198-w 33608571 PMC7896052

[40] Danis C, Dupré E, Zejneli O, Caillierez R, Arrial A, Bégard S, et al. Inhibition of Tau seeding by targeting Tau nucleation core within neurons with a single domain antibody fragment. Mol Ther. 2022;30(4):1484–99. Epub 20220107. doi: 10.1016/j.ymthe.2022.01.009 . 35007758 PMC9077319

[41] Dupré E, Danis C, Arrial A, Hanoulle X, Homa M, Cantrelle FX, et al. Single Domain Antibody Fragments as New Tools for the Detection of Neuronal Tau Protein in Cells and in Mice Studies. ACS Chem Neurosci. 2019;10(9):3997–4006. Epub 20190819. doi: 10.1021/acschemneuro.9b00217 . 31380615

[42] Muruganandam A, Tanha J, Narang S, Stanimirovic D. Selection of phage-displayed llama single-domain antibodies that transmigrate across human blood-brain barrier endothelium. Faseb j. 2002;16(2):240–2. Epub 20011228. doi: 10.1096/fj.01-0343fje . 11772942

[43] Farrington GK, Caram-Salas N, Haqqani AS, Brunette E, Eldredge J, Pepinsky B, et al. A novel platform for engineering blood-brain barrier-crossing bispecific biologics. Faseb j. 2014;28(11):4764–78. Epub 20140728. doi: 10.1096/fj.14-253369 . 25070367

[44] Wouters Y, Jaspers T, De Strooper B, Dewilde M. Identification and in vivo characterization of a brain-penetrating nanobody. Fluids Barriers CNS. 2020;17(1):62. Epub 20201014. doi: 10.1186/s12987-020-00226-z ; PubMed Central PMCID: PMC7556960. 33054787 PMC7556960

[45] Stanimirovic DKK, K.; Haqqani, A.S.; Sulea, T.; Arbabi-Ghahroudi, M.; Massie, B.; Gilbert, R., inventorInsulin-like growth factor 1 receptor-specific antibodies and uses thereof patent US10100117B2. 2018.

[46] Yogi A, Hussack G, van Faassen H, Haqqani AS, Delaney CE, Brunette E, et al. Brain Delivery of IGF1R5, a Single-Domain Antibody Targeting Insulin-like Growth Factor-1 Receptor. Pharmaceutics. 2022;14(7):1452. doi: 10.3390/pharmaceutics14071452 35890347 PMC9316817

[47] Stocki P, Szary J, Rasmussen CLM, Demydchuk M, Northall L, Logan DB, et al. Blood-brain barrier transport using a high affinity, brain-selective VNAR antibody targeting transferrin receptor 1. Faseb j. 2021;35(2):e21172. Epub 20201125. doi: 10.1096/fj.202001787R . 33241587

[48] Wu D, Yang J, Pardridge WM. Drug targeting of a peptide radiopharmaceutical through the primate blood-brain barrier in vivo with a monoclonal antibody to the human insulin receptor. J Clin Invest. 1997;100(7):1804–12. doi: 10.1172/JCI119708 ; PubMed Central PMCID: PMC508366. 9312181 PMC508366

[49] Garberg P, Ball M, Borg N, Cecchelli R, Fenart L, Hurst RD, et al. In vitro models for the blood-brain barrier. Toxicol In Vitro. 2005;19(3):299–334. doi: 10.1016/j.tiv.2004.06.011 . 15713540

[50] Carraway R, Leeman SE. The isolation of a new hypotensive peptide, neurotensin, from bovine hypothalami. J Biol Chem. 1973;248(19):6854–61. . 4745447

[51] Ratner C, Skov LJ, Raida Z, Bächler T, Bellmann-Sickert K, Le Foll C, et al. Effects of Peripheral Neurotensin on Appetite Regulation and Its Role in Gastric Bypass Surgery. Endocrinology. 2016;157(9):3482–92. Epub 20160720. doi: 10.1210/en.2016-1329 . 27580810

[52] Martin GE, Bacino CB, Papp NL. Hypothermia elicited by the intracerebral microinjection of neurotensin. Peptides. 1980;1(4):333–9. doi: 10.1016/0196-9781(80)90011-x . 7301636

[53] Kokko KP, Hadden MK, Price KL, Orwig KS, See RE, Dix TA. In vivo behavioral effects of stable, receptor-selective neurotensin[8–13] analogues that cross the blood-brain barrier. Neuropharmacology. 2005;48(3):417–25. doi: 10.1016/j.neuropharm.2004.10.008 . 15721174

[54] Young WS 3rd, Kuhar MJ. Neurotensin receptor localization by light microscopic autoradiography in rat brain. Brain Res. 1981;206(2):273–85. doi: 10.1016/0006-8993(81)90532-1 . 6260280

[55] Bissette G, Nemeroff CB, Loosen PT, Prange AJ, Lipton MA. Hypothermia and intolerance to cold induced by intracisternal administration of the hypothalamic peptide neurotensin. Nature. 1976;262(5569):607–9. doi: 10.1038/262607a0 8728

[56] Esparza TJ, Martin NP, Anderson GP, Goldman ER, Brody DL. High affinity nanobodies block SARS-CoV-2 spike receptor binding domain interaction with human angiotensin converting enzyme. Scientific Reports. 2020;10(1):22370. doi: 10.1038/s41598-020-79036-0 33353972 PMC7755911

[57] Pardon E, Laeremans T, Triest S, Rasmussen SGF, Wohlkönig A, Ruf A, et al. A general protocol for the generation of Nanobodies for structural biology. Nature Protocols. 2014;9(3):674–93. doi: 10.1038/nprot.2014.039 24577359 PMC4297639

[58] Paraschiv G, Vincke C, Czaplewska P, Manea M, Muyldermans S, Przybylski M. Epitope structure and binding affinity of single chain llama anti-β-amyloid antibodies revealed by proteolytic excision affinity-mass spectrometry. J Mol Recognit. 2013;26(1):1–9. doi: 10.1002/jmr.2210 . 23280612

[59] Martin AC. Accessing the Kabat antibody sequence database by computer. Proteins. 1996;25(1):130–3. Epub 1996/05/01. doi: 10.1002/(SICI)1097-0134(199605)25:1<130::AID-PROT11>3.0.CO;2-L . 8727325

[60] Quan S, Hiniker A, Collet JF, Bardwell JC. Isolation of bacteria envelope proteins. Methods Mol Biol. 2013;966:359–66. Epub 2013/01/10. doi: 10.1007/978-1-62703-245-2_22 . 23299746

[61] Jankowsky JL, Fadale DJ, Anderson J, Xu GM, Gonzales V, Jenkins NA, et al. Mutant presenilins specifically elevate the levels of the 42 residue beta-amyloid peptide in vivo: evidence for augmentation of a 42-specific gamma secretase. Hum Mol Genet. 2004;13(2):159–70. Epub 20031125. doi: 10.1093/hmg/ddh019 . 14645205

[62] Jankowsky JL, Slunt HH, Ratovitski T, Jenkins NA, Copeland NG, Borchelt DR. Co-expression of multiple transgenes in mouse CNS: a comparison of strategies. Biomol Eng. 2001;17(6):157–65. doi: 10.1016/s1389-0344(01)00067-3 . 11337275

[63] Styren SD, Hamilton RL, Styren GC, Klunk WE. X-34, a fluorescent derivative of Congo red: a novel histochemical stain for Alzheimer’s disease pathology. J Histochem Cytochem. 2000;48(9):1223–32. doi: 10.1177/002215540004800906 . 10950879

[64] Mamat U, Wilke K, Bramhill D, Schromm AB, Lindner B, Kohl TA, et al. Detoxifying Escherichia coli for endotoxin-free production of recombinant proteins. Microbial Cell Factories. 2015;14(1):57. doi: 10.1186/s12934-015-0241-5 25890161 PMC4404585

[65] Varatharaj A, Galea I. The blood-brain barrier in systemic inflammation. Brain Behav Immun. 2017;60:1–12. Epub 20160316. doi: 10.1016/j.bbi.2016.03.010 . 26995317

[66] Food and Drug Administration. Bacterial Endotoxins/Pyrogens. https://www.fda.gov/inspections-compliance-enforcement-and-criminal-investigations/inspection-technical-guides/bacterial-endotoxinspyrogens. November 2014.

[67] Schröter C, Günther R, Rhiel L, Becker S, Toleikis L, Doerner A, et al. A generic approach to engineer antibody pH-switches using combinatorial histidine scanning libraries and yeast display. MAbs. 2015;7(1):138–51. doi: 10.4161/19420862.2014.985993 ; PubMed Central PMCID: PMC4622719. 25523975 PMC4622719

[68] Maeda K, Kato Y, Sugiyama Y. pH-dependent receptor/ligand dissociation as a determining factor for intracellular sorting of ligands for epidermal growth factor receptors in rat hepatocytes. J Control Release. 2002;82(1):71–82. doi: 10.1016/s0168-3659(02)00126-8 . 12106978

[69] Tyler-McMahon BM, Boules M, Richelson E. Neurotensin: peptide for the next millennium. Regul Pept. 2000;93(1–3):125–36. doi: 10.1016/s0167-0115(00)00183-x . 11033059

[70] Broadwell RD, Balin BJ, Salcman M, Kaplan RS. Brain-blood barrier? Yes and no. Proc Natl Acad Sci U S A. 1983;80(23):7352–6. doi: 10.1073/pnas.80.23.7352 ; PubMed Central PMCID: PMC390053. 6580650 PMC390053

[71] Rodríguez EM, Blázquez JL, Guerra M. The design of barriers in the hypothalamus allows the median eminence and the arcuate nucleus to enjoy private milieus: the former opens to the portal blood and the latter to the cerebrospinal fluid. Peptides. 2010;31(4):757–76. Epub 20100120. doi: 10.1016/j.peptides.2010.01.003 . 20093161

[72] Morrison SF. Central control of body temperature. F1000Res. 2016;5. Epub 20160512. doi: 10.12688/f1000research.7958.1 ; PubMed Central PMCID: PMC4870994. 27239289 PMC4870994

[73] Yoo S, Blackshaw S. Regulation and function of neurogenesis in the adult mammalian hypothalamus. Prog Neurobiol. 2018;170:53–66. Epub 20180406. doi: 10.1016/j.pneurobio.2018.04.001 ; PubMed Central PMCID: PMC6173995. 29631023 PMC6173995

[74] Cheunsuang O, Stewart AL, Morris R. Differential uptake of molecules from the circulation and CSF reveals regional and cellular specialisation in CNS detection of homeostatic signals. Cell and Tissue Research. 2006;325(2):397–402. doi: 10.1007/s00441-006-0162-z 16555054

[75] Haddad-Tóvolli R, Dragano NRV, Ramalho AFS, Velloso LA. Development and Function of the Blood-Brain Barrier in the Context of Metabolic Control. Front Neurosci. 2017;11:224. Epub 20170421. doi: 10.3389/fnins.2017.00224 ; PubMed Central PMCID: PMC5399017. 28484368 PMC5399017

[76] Morita S, Miyata S. Accessibility of low-molecular-mass molecules to the median eminence and arcuate hypothalamic nucleus of adult mouse. Cell Biochem Funct. 2013;31(8):668–77. Epub 20130124. doi: 10.1002/cbf.2953 . 23348371

[77] Triguero D, Buciak J, Pardridge WM. Capillary depletion method for quantification of blood-brain barrier transport of circulating peptides and plasma proteins. J Neurochem. 1990;54(6):1882–8. doi: 10.1111/j.1471-4159.1990.tb04886.x . 2338547

[78] Moos T, Morgan EH. Transferrin and Transferrin Receptor Function in Brain Barrier Systems. Cellular and Molecular Neurobiology. 2000;20(1):77–95. doi: 10.1023/a:1006948027674 10690503 PMC11537550

[79] Niewoehner J, Bohrmann B, Collin L, Urich E, Sade H, Maier P, et al. Increased brain penetration and potency of a therapeutic antibody using a monovalent molecular shuttle. Neuron. 2014;81(1):49–60. doi: 10.1016/j.neuron.2013.10.061 . 24411731

[80] Boado RJ, Zhang Y, Wang Y, Pardridge WM. Engineering and expression of a chimeric transferrin receptor monoclonal antibody for blood-brain barrier delivery in the mouse. Biotechnol Bioeng. 2009;102(4):1251–8. doi: 10.1002/bit.22135 ; PubMed Central PMCID: PMC2729652. 18942151 PMC2729652

[81] Kariolis MS, Wells RC, Getz JA, Kwan W, Mahon CS, Tong R, et al. Brain delivery of therapeutic proteins using an Fc fragment blood-brain barrier transport vehicle in mice and monkeys. Sci Transl Med. 2020;12(545). doi: 10.1126/scitranslmed.aay1359 . 32461332

[82] Lee HJ, Engelhardt B, Lesley J, Bickel U, Pardridge WM. Targeting rat anti-mouse transferrin receptor monoclonal antibodies through blood-brain barrier in mouse. J Pharmacol Exp Ther. 2000;292(3):1048–52. . 10688622

[83] Chen C, Liang Z, Zhou B, Li X, Lui C, Ip NY, et al. In Vivo Near-Infrared Two-Photon Imaging of Amyloid Plaques in Deep Brain of Alzheimer’s Disease Mouse Model. ACS Chem Neurosci. 2018;9(12):3128–36. Epub 20180816. doi: 10.1021/acschemneuro.8b00306 . 30067906

[84] Kamphuis W, Mamber C, Moeton M, Kooijman L, Sluijs JA, Jansen AH, et al. GFAP isoforms in adult mouse brain with a focus on neurogenic astrocytes and reactive astrogliosis in mouse models of Alzheimer disease. PLoS One. 2012;7(8):e42823. Epub 20120813. doi: 10.1371/journal.pone.0042823 ; PubMed Central PMCID: PMC3418292. 22912745 PMC3418292

[85] Cecchelli R, Aday S, Sevin E, Almeida C, Culot M, Dehouck L, et al. A stable and reproducible human blood-brain barrier model derived from hematopoietic stem cells. PLoS One. 2014;9(6):e99733. Epub 20140617. doi: 10.1371/journal.pone.0099733 ; PubMed Central PMCID: PMC4061029. 24936790 PMC4061029

[86] Shayan G, Choi YS, Shusta EV, Shuler ML, Lee KH. Murine in vitro model of the blood-brain barrier for evaluating drug transport. Eur J Pharm Sci. 2011;42(1–2):148–55. Epub 20101113. doi: 10.1016/j.ejps.2010.11.005 . 21078386

[87] Stanimirovic DB, Bani-Yaghoub M, Perkins M, Haqqani AS. Blood-brain barrier models: in vitro to in vivo translation in preclinical development of CNS-targeting biotherapeutics. Expert Opin Drug Discov. 2015;10(2):141–55. Epub 20141112. doi: 10.1517/17460441.2015.974545 . 25388782

[88] Couch JA, Yu YJ, Zhang Y, Tarrant JM, Fuji RN, Meilandt WJ, et al. Addressing safety liabilities of TfR bispecific antibodies that cross the blood-brain barrier. Sci Transl Med. 2013;5(183):183ra57, 1–12. doi: 10.1126/scitranslmed.3005338 . 23636093

[89] Pardridge WM. A Historical Review of Brain Drug Delivery. Pharmaceutics. 2022;14(6):1283. doi: 10.3390/pharmaceutics14061283 35745855 PMC9229021

[90] Gadkar K, Yadav DB, Zuchero JY, Couch JA, Kanodia J, Kenrick MK, et al. Mathematical PKPD and safety model of bispecific TfR/BACE1 antibodies for the optimization of antibody uptake in brain. Eur J Pharm Biopharm. 2016;101:53–61. Epub 2016/01/29. doi: 10.1016/j.ejpb.2016.01.009 . 26820920

[91] Gorlani A, Brouwers J, McConville C, van der Bijl P, Malcolm K, Augustijns P, et al. Llama antibody fragments have good potential for application as HIV type 1 topical microbicides. AIDS Res Hum Retroviruses. 2012;28(2):198–205. Epub 20110824. doi: 10.1089/aid.2011.0133 . 21864083

[92] Su S, Esparza TJ, Nguyen D, Mastrogiacomo S, Kim JH, Brody DL. Pharmacokinetics of Single Domain Antibodies and Conjugated Nanoparticles Using a Hybrid near Infrared Method. Int J Mol Sci. 2021;22(16). Epub 20210813. doi: 10.3390/ijms22168695 ; PubMed Central PMCID: PMC8395466. 34445399 PMC8395466

[93] van Faassen H, Ryan S, Henry KA, Raphael S, Yang Q, Rossotti MA, et al. Serum albumin-binding V(H) Hs with variable pH sensitivities enable tailored half-life extension of biologics. Faseb j. 2020;34(6):8155–71. Epub 20200428. doi: 10.1096/fj.201903231R . 32342547

[94] Rotman M, Welling MM, van den Boogaard ML, Moursel LG, van der Graaf LM, van Buchem MA, et al. Fusion of hIgG1-Fc to 111In-anti-amyloid single domain antibody fragment VHH-pa2H prolongs blood residential time in APP/PS1 mice but does not increase brain uptake. Nucl Med Biol. 2015;42(8):695–702. Epub 20150318. doi: 10.1016/j.nucmedbio.2015.03.003 . 25960433

[95] Li Q, Barrett A, Vijayakrishnan B, Tiberghien A, Beard R, Rickert KW, et al. Improved Inhibition of Tumor Growth by Diabody-Drug Conjugates via Half-Life Extension. Bioconjug Chem. 2019;30(4):1232–43. Epub 20190403. doi: 10.1021/acs.bioconjchem.9b00170 . 30912649

[96] Lord B, Ameriks MK, Wang Q, Fourgeaud L, Vliegen M, Verluyten W, et al. A novel radioligand for the ATP-gated ion channel P2X7: [3H] JNJ-54232334. Eur J Pharmacol. 2015;765:551–9. Epub 20150918. doi: 10.1016/j.ejphar.2015.09.026 . 26386289

[97] Basak I, Patil KS, Alves G, Larsen JP, Møller SG. microRNAs as neuroregulators, biomarkers and therapeutic agents in neurodegenerative diseases. Cell Mol Life Sci. 2016;73(4):811–27. Epub 2015/11/27. doi: 10.1007/s00018-015-2093-x . 26608596 PMC11108480

[98] Koikkalainen J, Rhodius-Meester H, Tolonen A, Barkhof F, Tijms B, Lemstra AW, et al. Differential diagnosis of neurodegenerative diseases using structural MRI data. Neuroimage Clin. 2016;11:435–49. Epub 2016/04/23. doi: 10.1016/j.nicl.2016.02.019 ; PubMed Central PMCID: PMC4827727. 27104138 PMC4827727

[99] Kim JH, Dodd S, Ye FQ, Knutsen AK, Nguyen D, Wu H, et al. Sensitive detection of extremely small iron oxide nanoparticles in living mice using MP2RAGE with advanced image co-registration. Scientific Reports. 2021;11(1):106. doi: 10.1038/s41598-020-80181-9 33420210 PMC7794370

[100] Liu XG, Lu S, Liu DQ, Zhang L, Zhang LX, Yu XL, et al. ScFv-conjugated superparamagnetic iron oxide nanoparticles for MRI-based diagnosis in transgenic mouse models of Parkinson’s and Huntington’s diseases. Brain Res. 2019;1707:141–53. Epub 20181124. doi: 10.1016/j.brainres.2018.11.034 . 30481502

[101] Sillerud LO, Solberg NO, Chamberlain R, Orlando RA, Heidrich JE, Brown DC, et al. SPION-enhanced magnetic resonance imaging of Alzheimer’s disease plaques in AβPP/PS-1 transgenic mouse brain. J Alzheimers Dis. 2013;34(2):349–65. Epub 2012/12/12. doi: 10.3233/JAD-121171 ; PubMed Central PMCID: PMC4801216. 23229079 PMC4801216

