# Supplementary figures and images for: Selection of single domain anti-transferrin receptor antibodies for blood-brain barrier transcytosis using a neurotensin based assay and histological assessment of target engagement in a mouse model of Alzheimer’s related amyloid-beta pathology
Source: PLoS One. 2022 Oct 18;17(10):e0276107. doi: 10.1371/journal.pone.0276107 (PMC9578589; doi:10.1371/journal.pone.0276107)

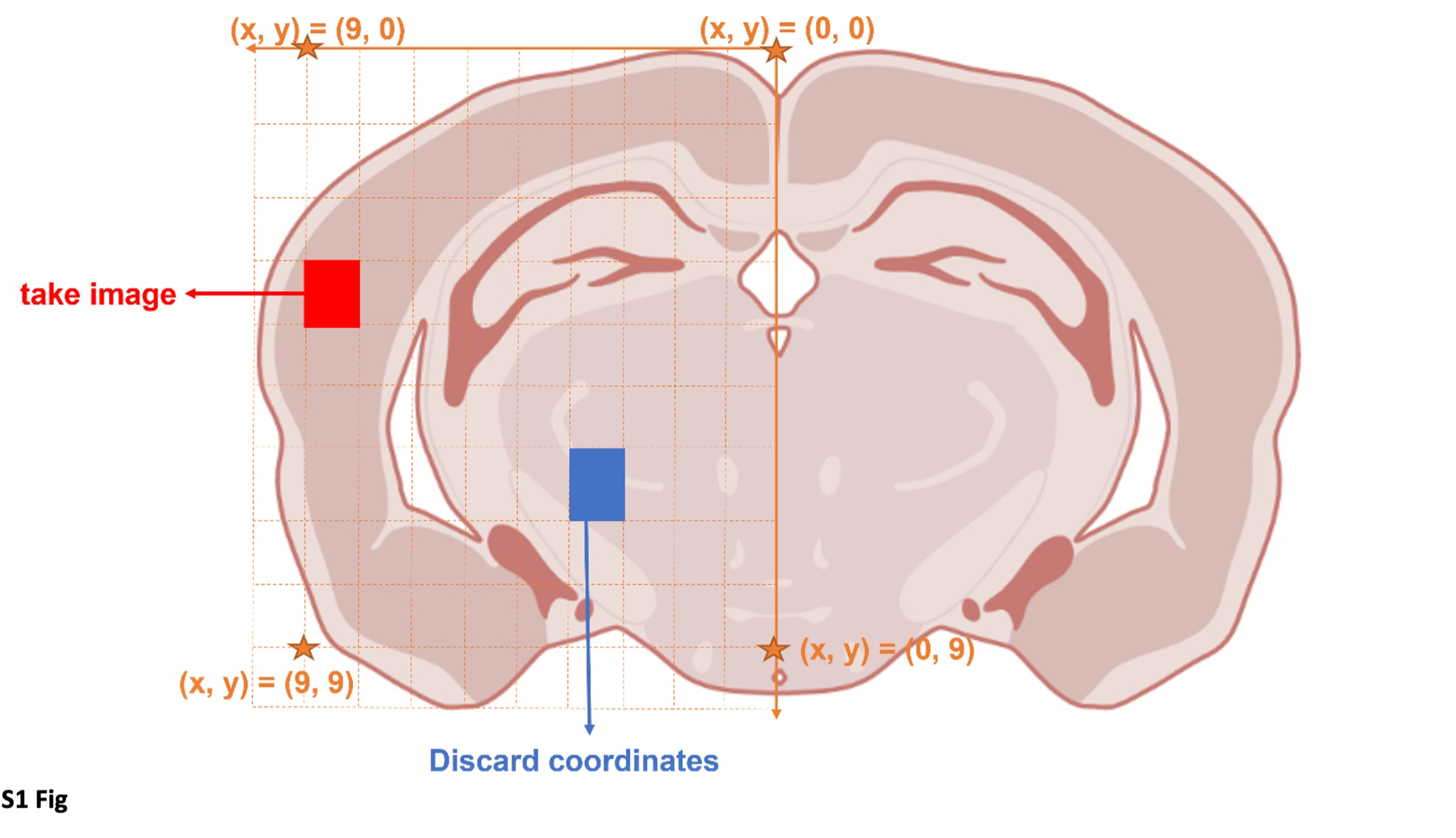

Supplement: S1 Fig — This schematic graph shows the logic of picking coordinates for confocal images acquisition. The right hemisphere of the brain was equally divided into ten parts in both x and y directions. (x, y) coordinates were randomly generated using a random number generator. The microscope stage was moved to the target (x, y) coordinates using the stage rulings. When (x, y) coordinates fell into the areas of cortex, images were taken and cropped to include only cortical areas. When (x, y) coordinates fell out of the cortex the coordinates were discarded and no images were taken. Source of the mouse brain: biorender.com. (TIF) [file pone.0276107.s001.tif]

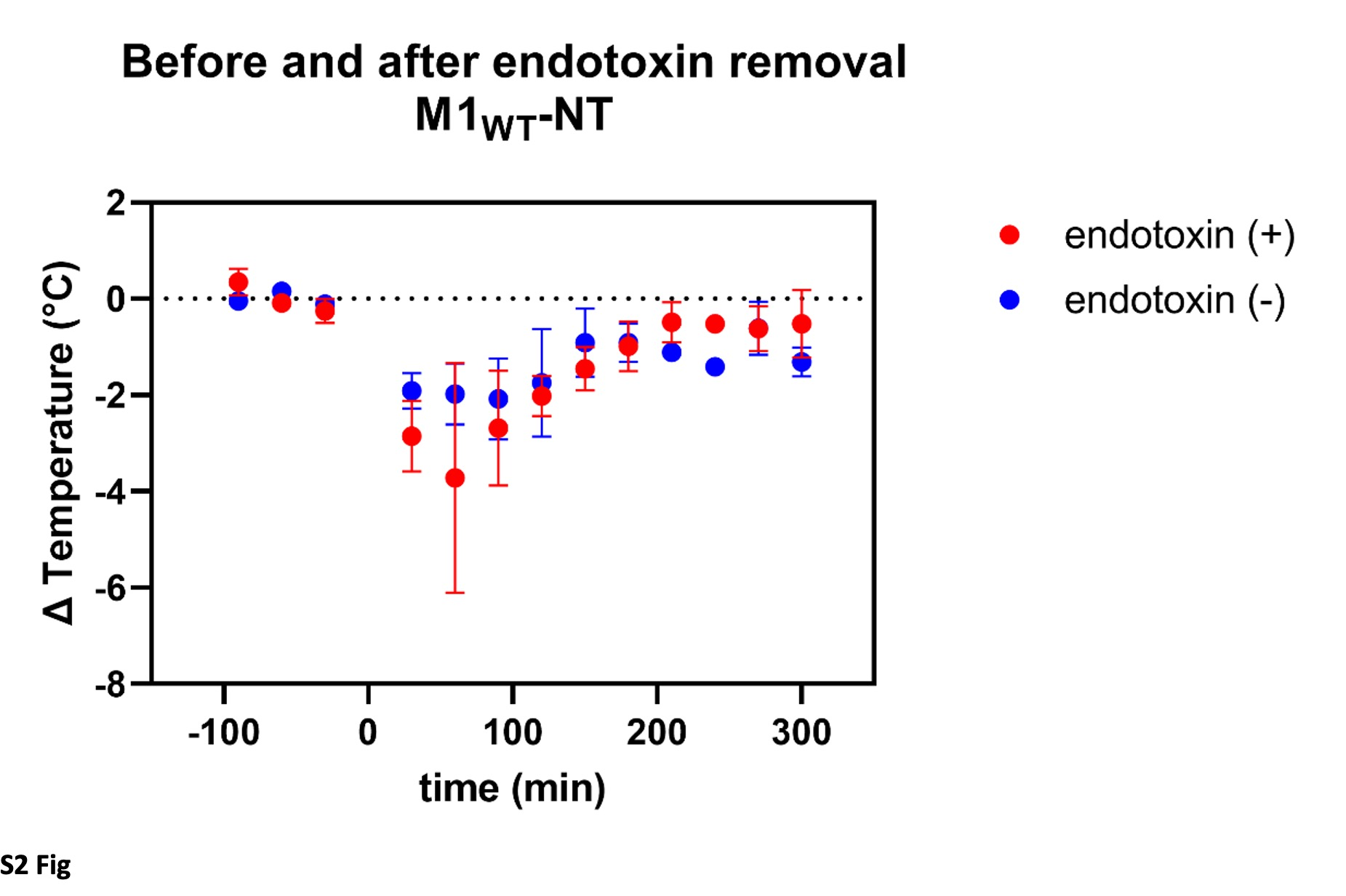

Supplement: S2 Fig — Endotoxin removal from in M1WT-NT preparations from E. coli decreased hypothermia effects at time 30min, 1hr and 1.5hr after IV injection of the M1WT -NT (n = 3 per group). The maximum hypothermic effect of 600nmol/kg body weight of M1WT -NT was approximately 2C° less after endotoxin removal. (TIF) [file pone.0276107.s002.tif]

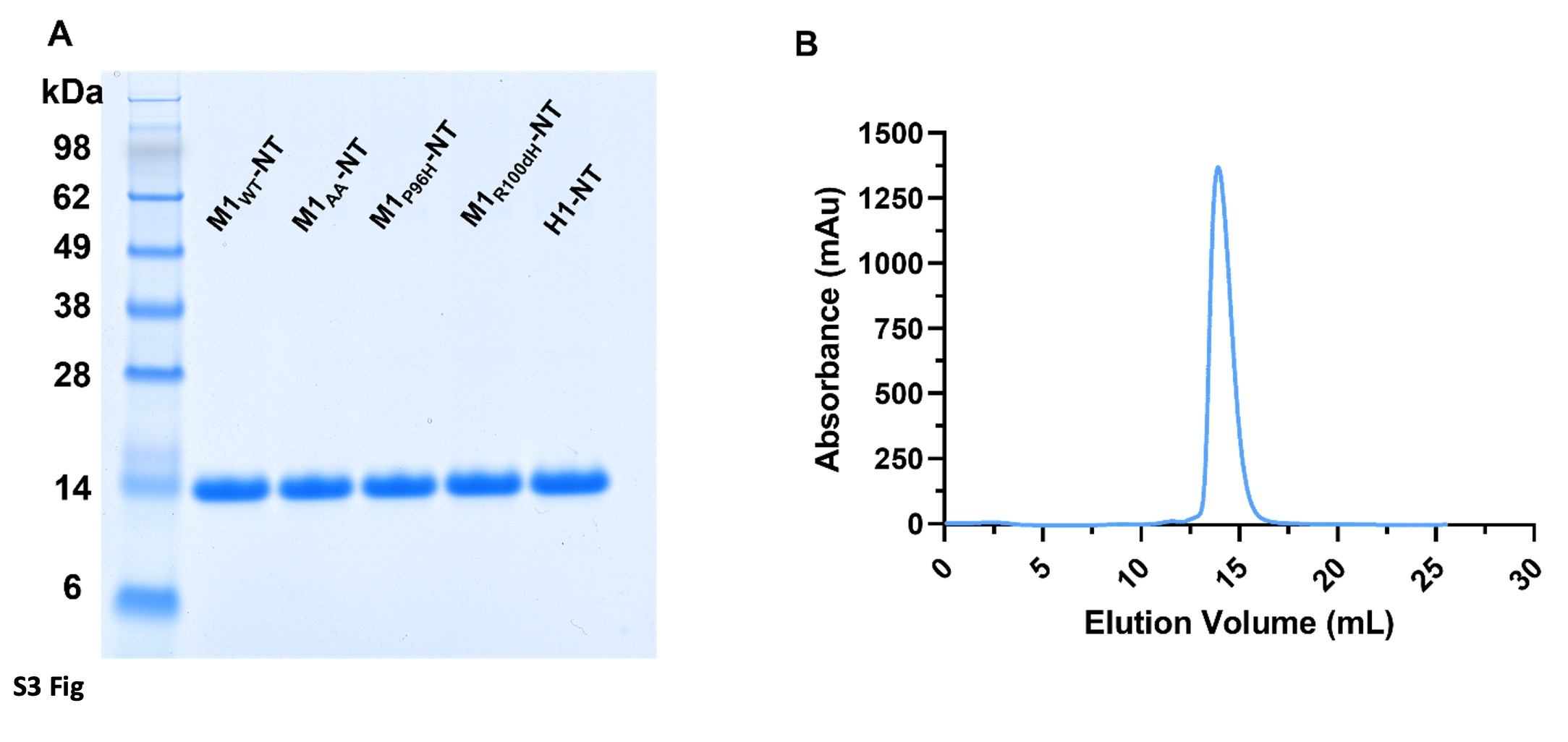

Supplement: S3 Fig — The purity of the monomer VHH by (a) SDS-PAGE gel and exemplar (b) size-exclusion chromatography over a Superdex75 column for M1WT-NT indicating >95% purity and homogeneity of the material. (TIF) [file pone.0276107.s003.tif]

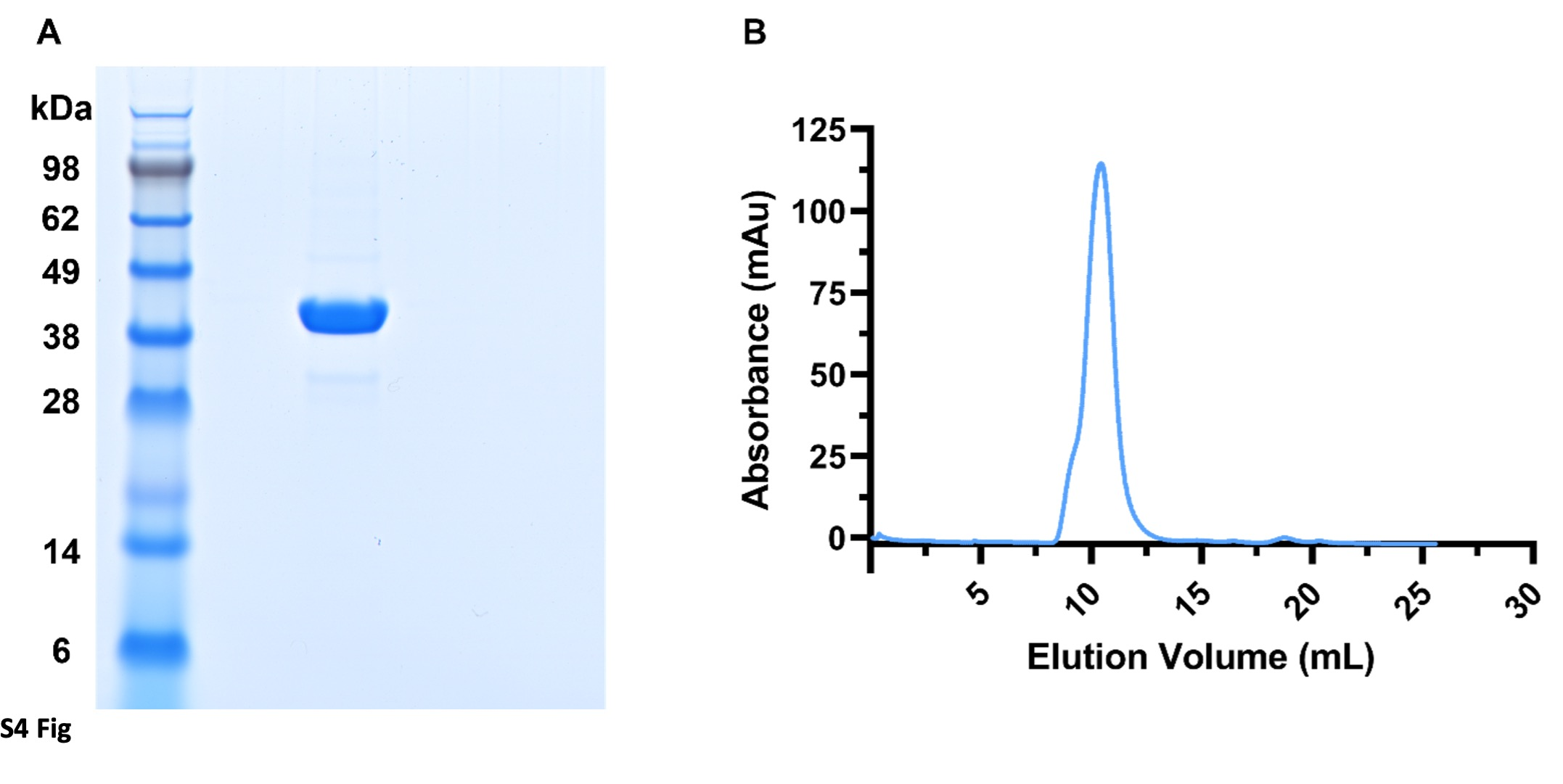

Supplement: S4 Fig — The purity of the triplet VHH by (a) SDS-PAGE gel and exemplar (b) size-exclusion chromatography over a Superdex75 column for M1P96H-Triplet-NT indicating >95% purity and homogeneity of the material. (TIF) [file pone.0276107.s004.tif]

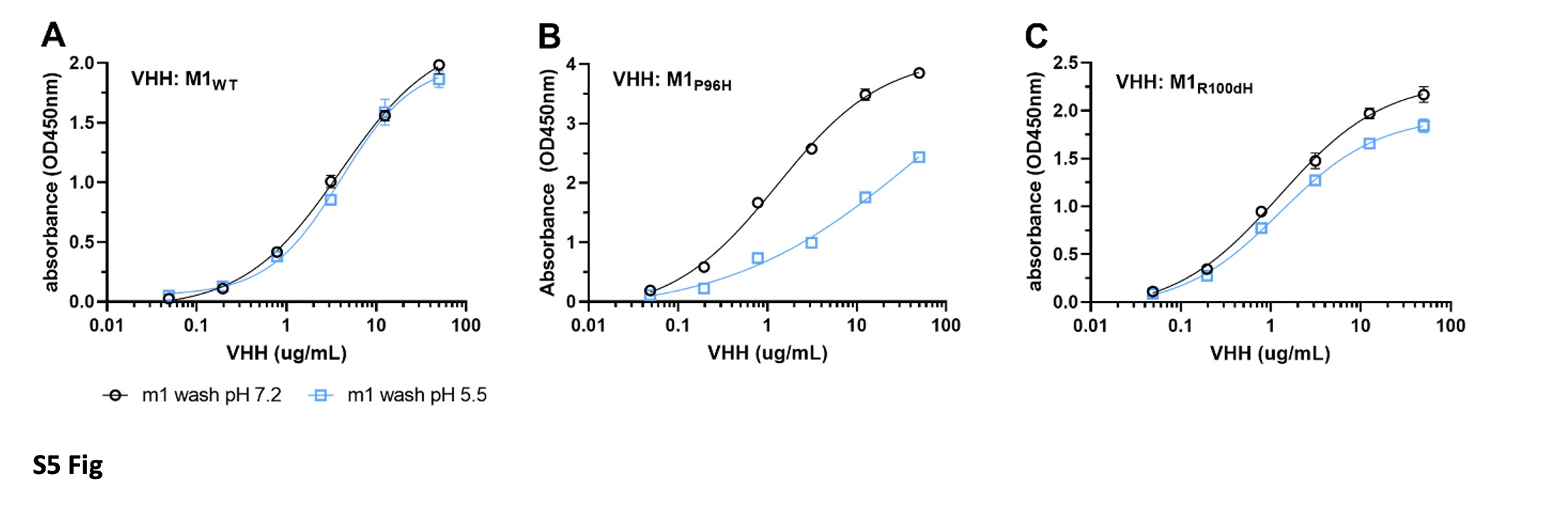

Supplement: S5 Fig — M1 variants (a) M1WT, (b) M1P96H and (c) M1R100dH were incubated on mTfR coated ELISA plates, followed by a stringent wash with 1x PBS buffer at pH 7.2 or pH 5.5. Following pH dependent washing, the bound VHH was detected with an anti-alpaca-peroxidase antibody and the reaction terminated by addition of 1M HCl. Error bars represent the standard deviation of the mean values at each data point. (TIF) [file pone.0276107.s005.tif]

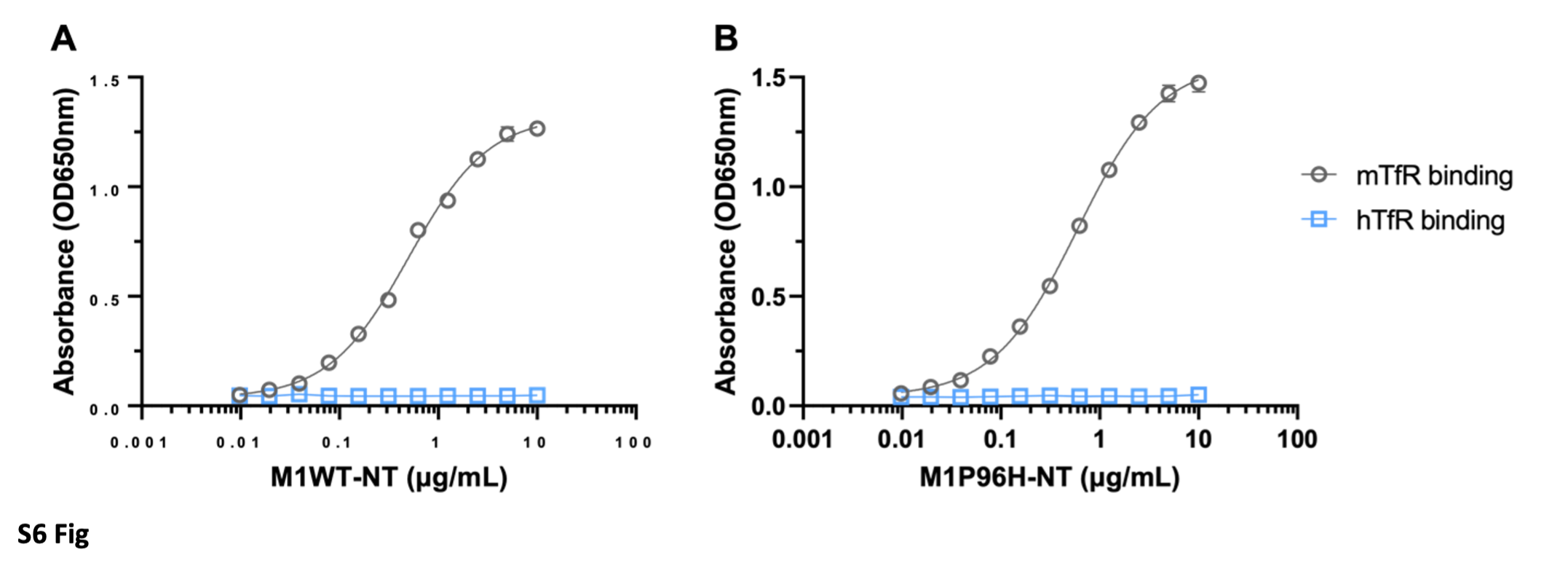

Supplement: S6 Fig — mTfR and hTfR ELISA on M1WT-NT (a) and M1P96H-NT (b). VHHs were incubated with mTfR or hTfR absorbed to ELISA plates and detected using an anti-alpaca-peroxidase antibody to determine the impact of fluorophore conjugation. The curves show no measurable binding of M1WT-NT (a) and M1P96H- NT (b) to human TfR extracellular domain. (TIF) [file pone.0276107.s006.tif]

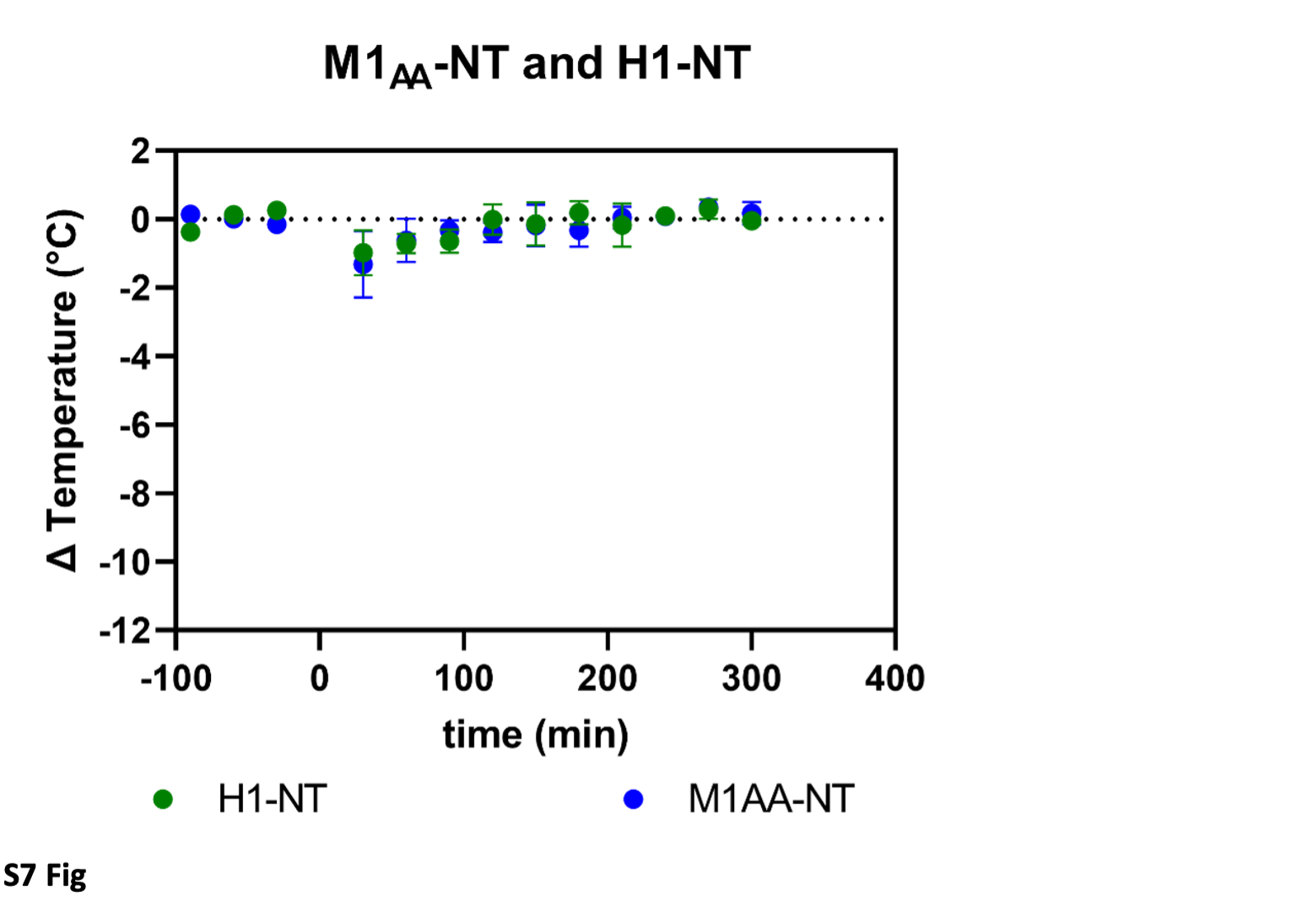

Supplement: S7 Fig — M1AA-NT and H1-NT were injected at doses of 1400nmol/kg body weight. M1AA-NT and H1-NT lack prominent hypothermia effects (n = 3 per group). (TIF) [file pone.0276107.s007.tif]

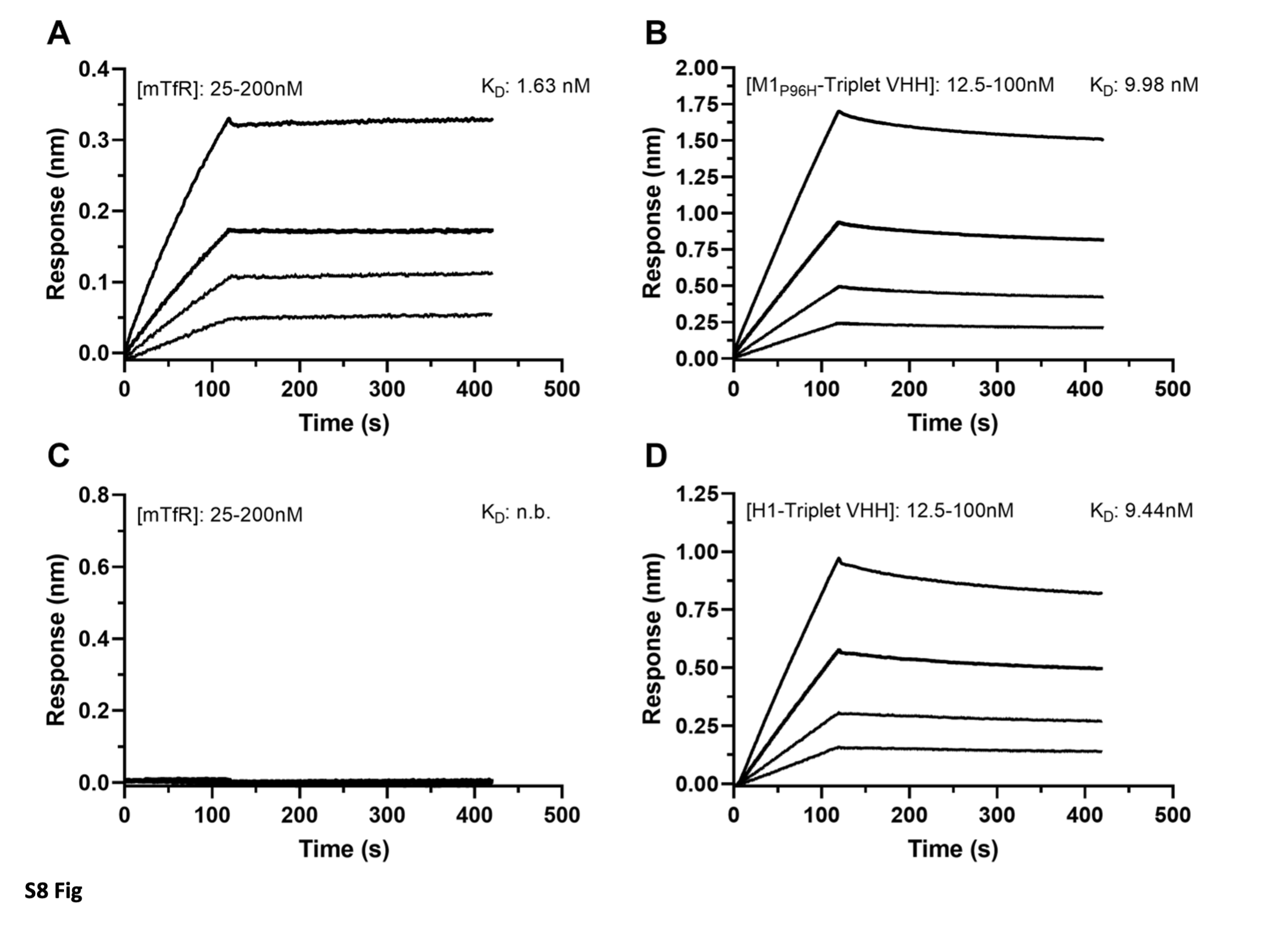

Supplement: S8 Fig — Using biolayer interferometry on an Octet Red96 system, association and dissociation rates were determined by immobilizing biotinylated VHH (a, c) or biotinylated amyloid beta (b, d) onto streptavidin-coated optical sensors. M1P96H-triplet-NT association and dissociation curve to (a) mTfR and (b) Aβ. The affinity of M1P96H-triplet-NTwas measured to be 1.63nM to mTfR and 9.98nM to Aβ. H1-triplet-NT association and dissociation curve to (c) mTfR and (d) Aβ. The affinity of H1-triplet-NT was measured to be 9.44nM to Aβ and no binding to mTfR. (n.b. = no binding). (TIF) [file pone.0276107.s008.tif]

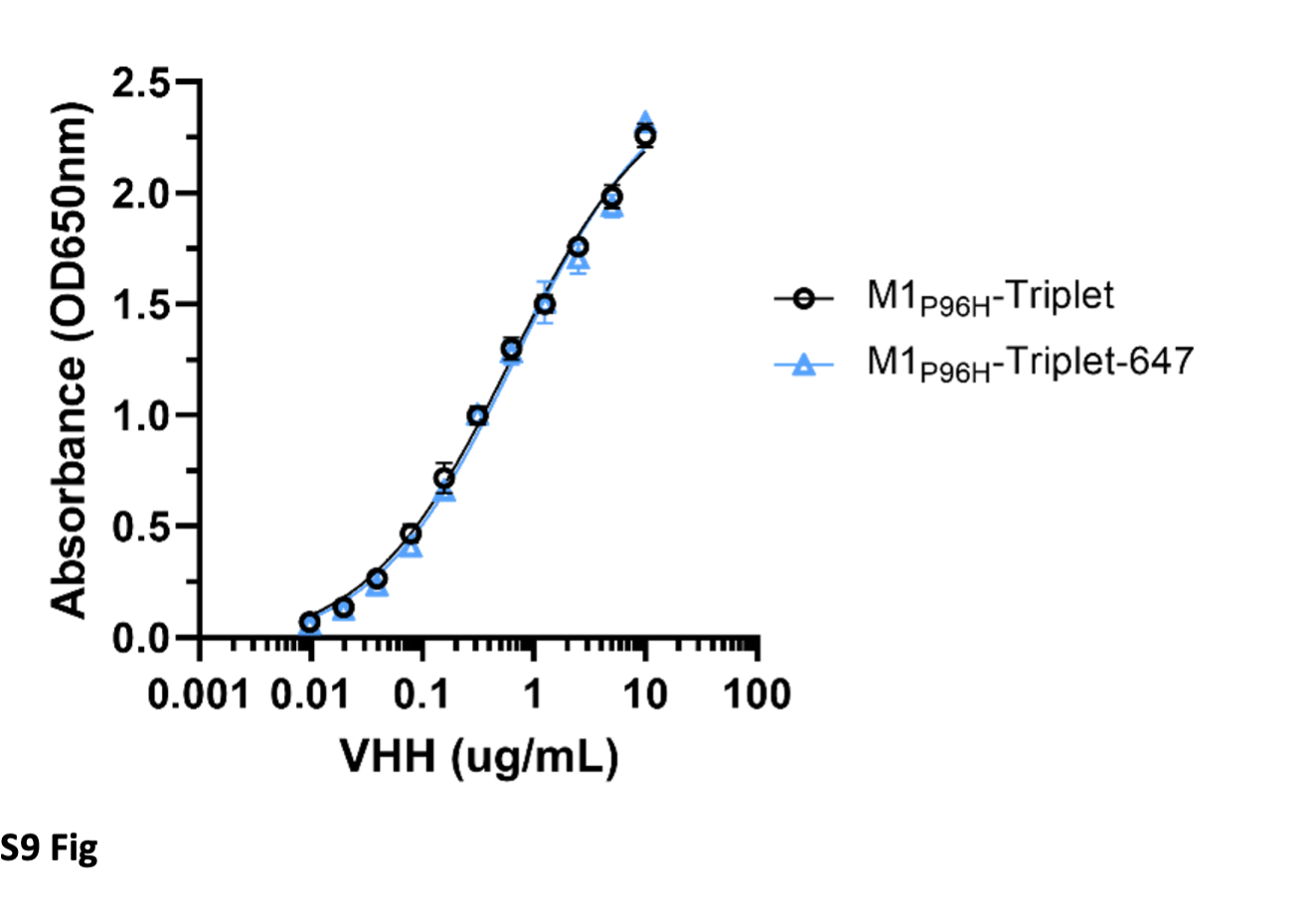

Supplement: S9 Fig — VHH was incubated with mTfR absorbed to ELISA plates and detected using an anti-alpaca-peroxidase antibody to determine the impact of fluorophore conjugation. The near overlapping binding curves indicate a lack of effect following fluorophore labelling. Error bars represent the standard deviation of the mean values at each data point. (TIF) [file pone.0276107.s009.tif]

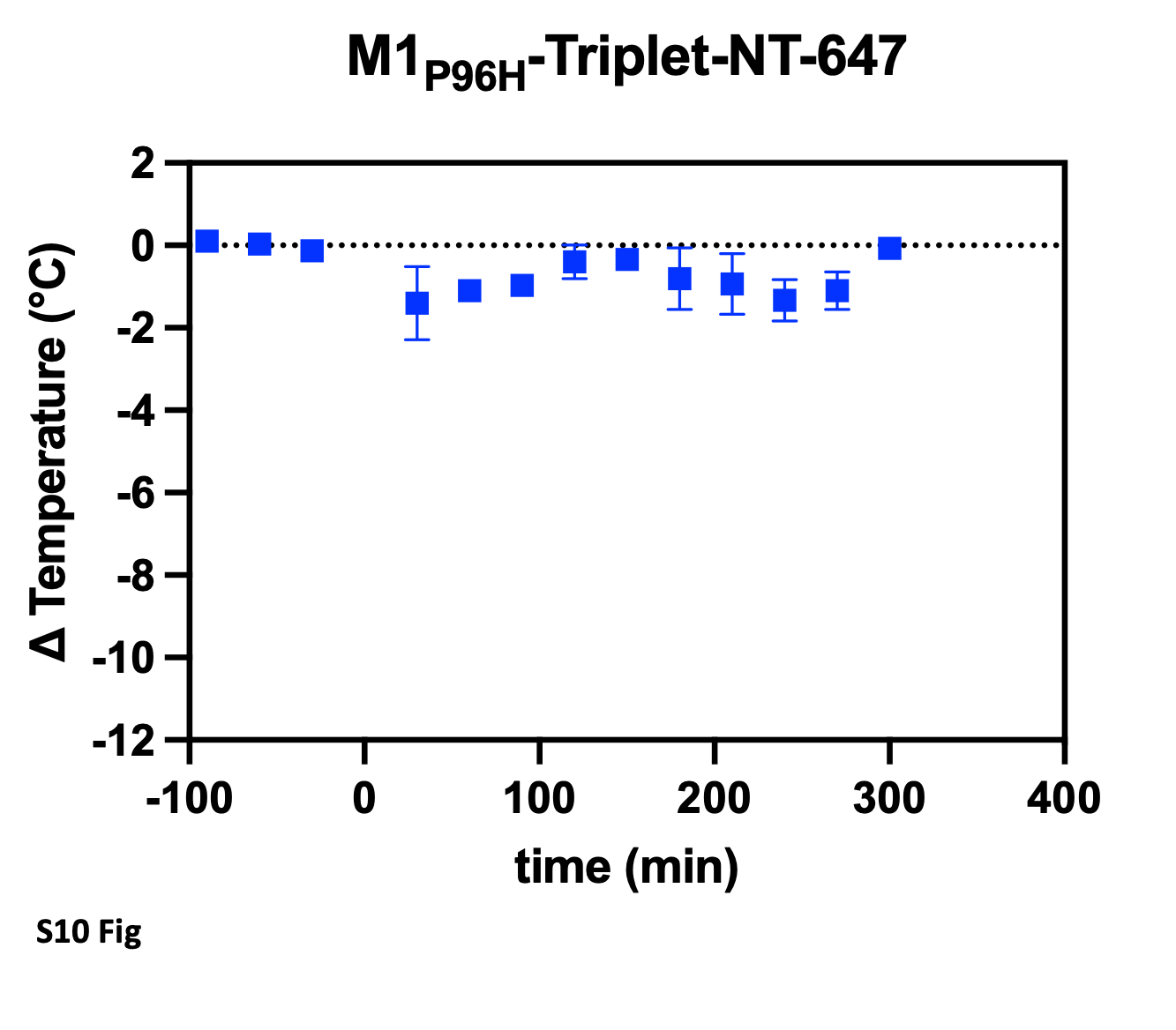

Supplement: S10 Fig — M1P96H-Triplet-NT-647 was injected at dose of 600nmol/kg body weight (n = 3). M1P96H-Triplet-NT-647 did not cause temperature drop. (TIF) [file pone.0276107.s010.tif]

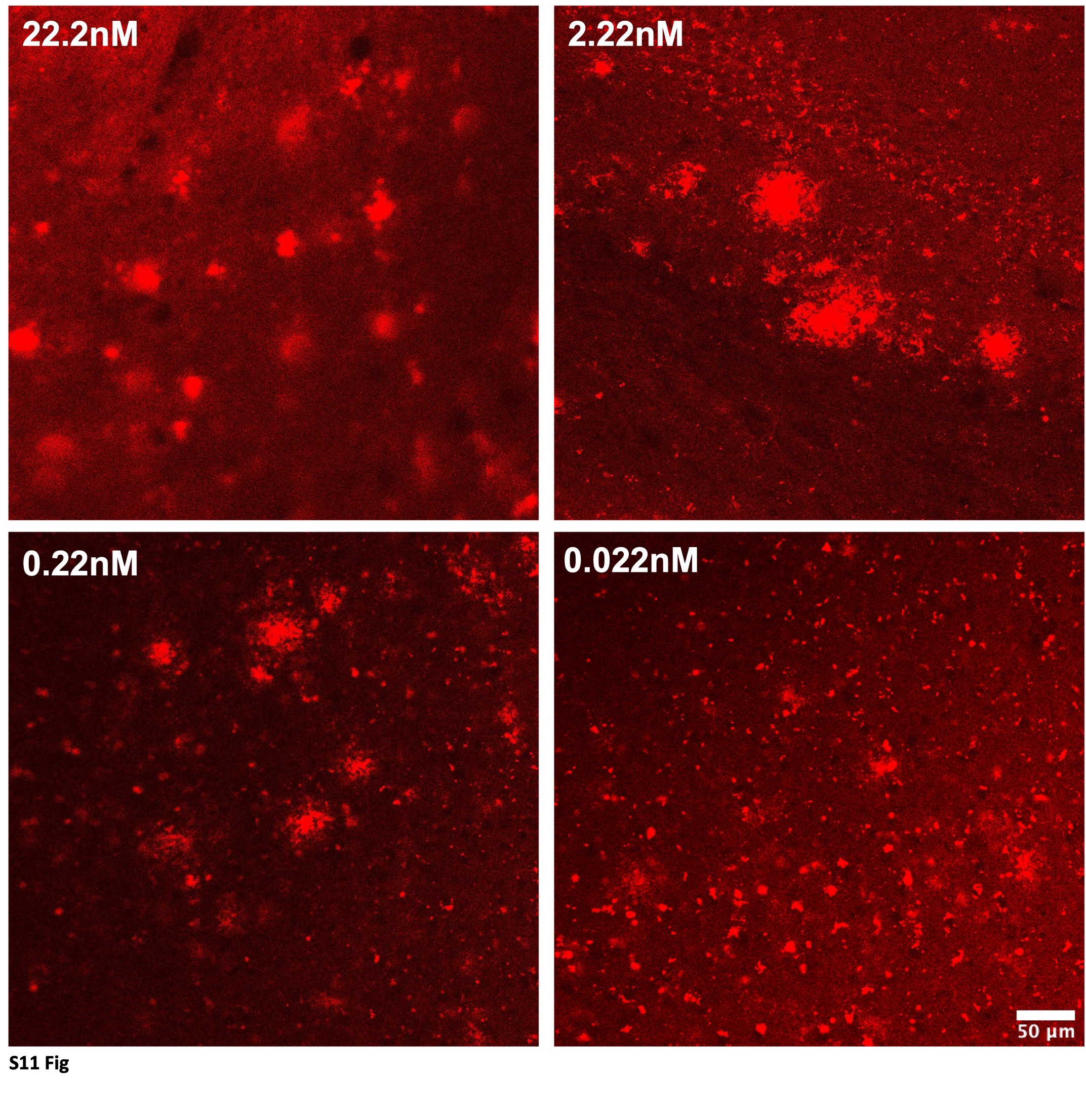

Supplement: S11 Fig — Mice brain sections were stained with M1P96H-Triplet-647 which labels the amyloid plaques. The representative 647 channel microscopy images of the ex vivo brain sections show substantial amyloid plaques labeling with M1P96H-Triplet-647 at concentration of 22.2nM, 2.22nM, and 0.22nM. (TIF) [file pone.0276107.s011.tif]

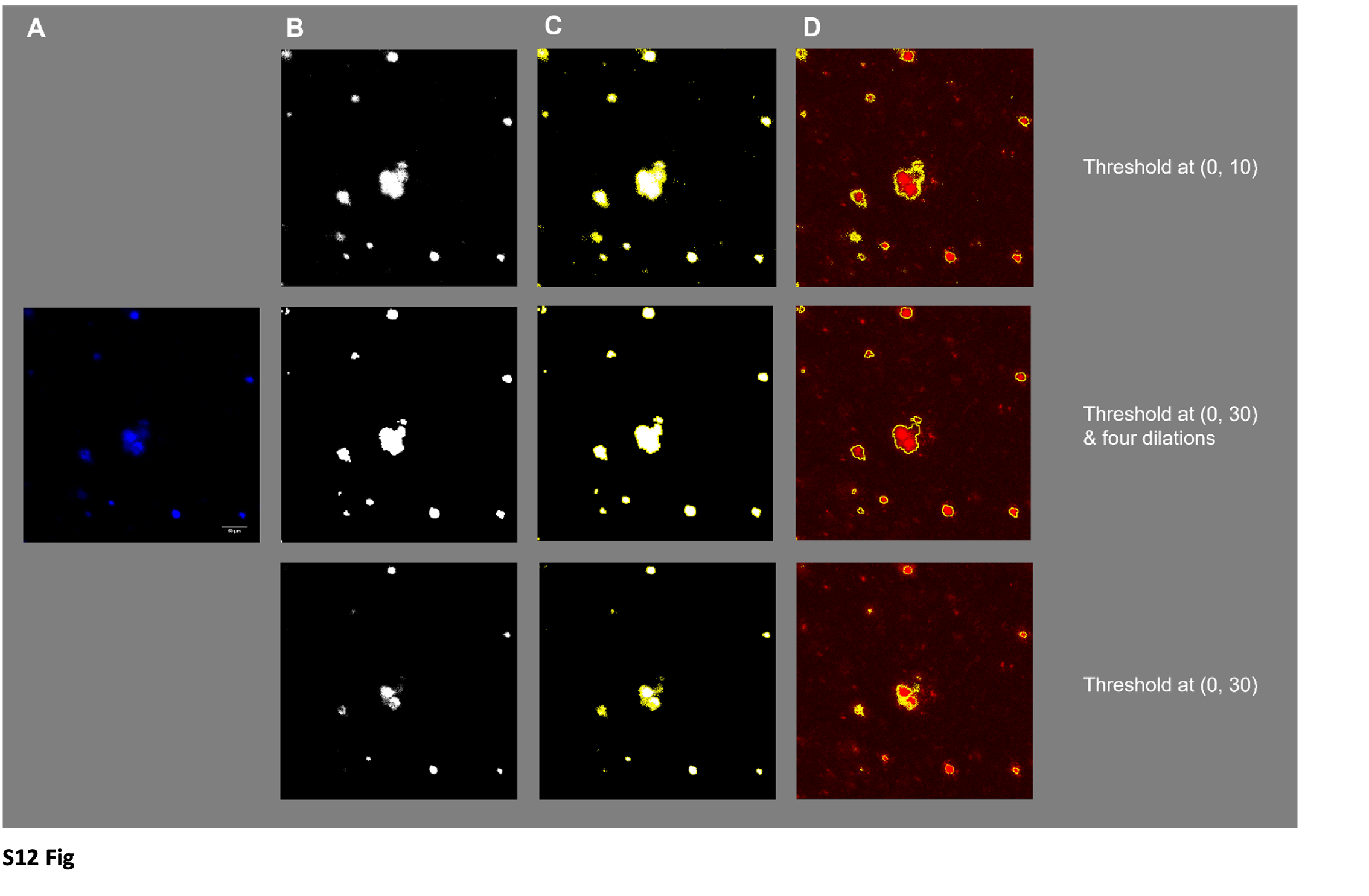

Supplement: S12 Fig — An APP/PS1 positive naïve brain slice with ex vivo M1P96H-Triplet-647 labeling was used as the example. Column a. shows a representative confocal microscope image of cortex X34 staining. Column b. shows the X34 labeled images after thresholding. Column c. shows automated selection of regions of plaques based on the thresholded images. Column d. shows the application of selected areas (from thresholded X34 images) to the 647 channel images. (TIF) [file pone.0276107.s012.tif]

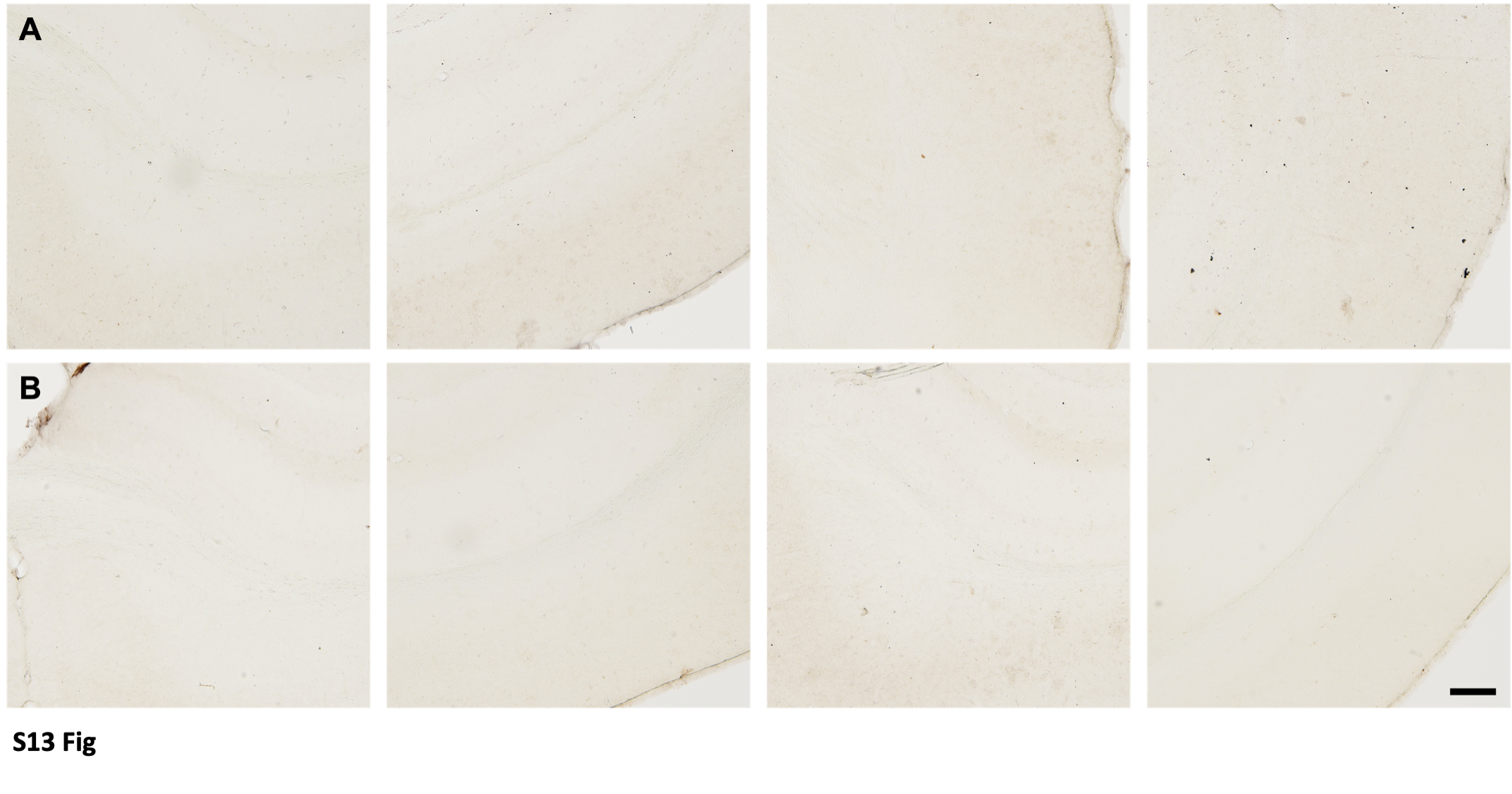

Supplement: S13 Fig — Anti-alpaca VHH secondary antibody against triplet-647 was used for immunohistochemistry. Mouse brains injected with H1-triplet-647 (a) and M1P96H-triplet-647 (b). Scale bar is 200 μm. (TIF) [file pone.0276107.s013.tif]

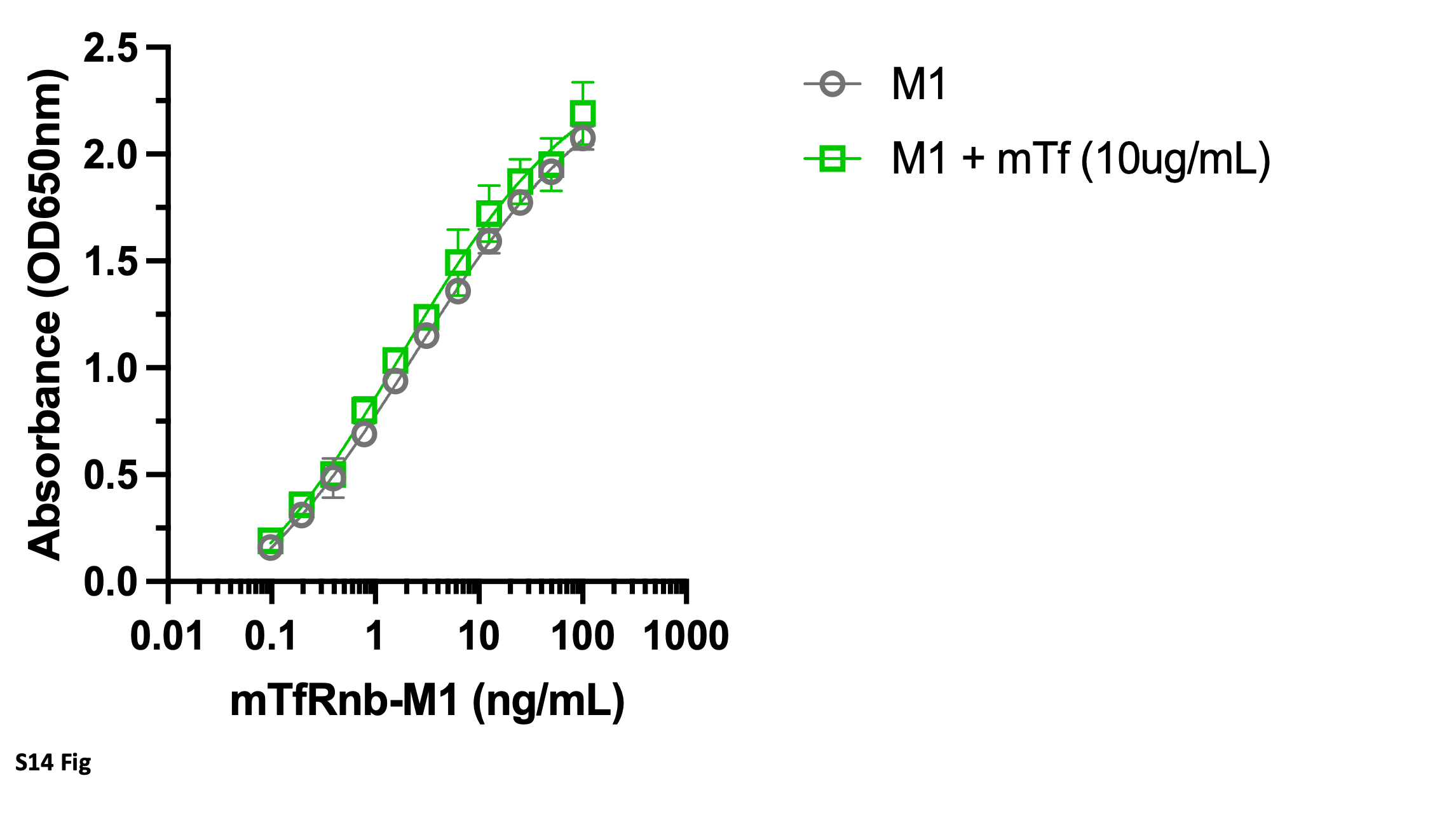

Supplement: S14 Fig — No change in binding affinity of mTfRnb-M1 to mTfR in the presence or absence of 10ug/ml of. Anti-mTfR VHH M1 was incubated with mTfR absorbed to ELISA plates and detected using an anti-alpaca-peroxidase antibody to determine the impact of mouse transferrin presence. The binding curves nearly overlap, indicating the presence or absence of mouse transferrin did not affect the binding affinity of M1 to mTfR. Error bars represent the standard deviation of the mean values at each data point. (TIF) [file pone.0276107.s014.tif]
