# Supplementary material for: Selection of single domain anti-transferrin receptor antibodies for blood-brain barrier transcytosis using a neurotensin based assay and histological assessment of target engagement in a mouse model of Alzheimer’s related amyloid-beta pathology
Source: PLoS One. 2022 Oct 18;17(10):e0276107. doi: 10.1371/journal.pone.0276107 (PMC9578589; doi:10.1371/journal.pone.0276107)
Supplement: S1 File — Staining was performed to visualize amyloid plaques using the Anti-alpaca VHH secondary nanobody. Free-floating 50 μm tissue was rinsed with PBS for three times. Then, tissues were incubated in 2% normal goat serum (NGS) with 0.1% Triton X-100 in PBS for 60min to block non-specific binding. After blocking, tissue was rinsed with 1X PBS for three times then incubated in 0.5% NGS with 0.1% Triton X-100 in PBS with 0.8mg/ml Anti-alpaca VHH secondary antibody (Jackson ImmunoResearch, 128-065-232) for 60min. After antibody incubation, the tissue was rinsed with PBS for three times, followed by incubation with ABC Elite (Vector Laboratories) at a 1:400 dilution in PBS for 60min. The tissue was then rinsed with PBS for three times and developed with 3,3’-diaminobenzidine (#D5905; Sigma-Aldrich). The tissue was rinsed with 1X PBS for three times then mounted onto positively charged slides (EF15978Z, Daigger®). The mounted tissue sections were allowed to dry overnight at room temperature and dehydrated using ethanol series. Then, the sections were cover slipped using VectaMount® Permanent Mounting Medium (H-5000-60). (PDF) [file pone.0276107.s015.pdf]

S1 Supporting Information. Alternative Light Microscopic Detection. Staining was performed to visualize amyloid plaques using the Anti-alpaca VHH secondary nanobody. Free-floating 50 µm tissue was rinsed with PBS for three times. Then, tissues were incubated in 2% normal goat serum (NGS) with 0.1% Triton X-100 in PBS for 60min to block non-specific binding. After blocking, tissue was rinsed with 1X PBS for three times then incubated in 0.5% NGS with 0.1% Triton X-100 in PBS with 0.8mg/ml Anti-alpaca VHH secondary antibody (Jackson ImmunoResearch, 128-065-232) for 60min. After antibody incubation, the tissue was rinsed with PBS for three times, followed by incubation with ABC Elite (Vector Laboratories) at a 1:400 dilution in PBS for 60min. The tissue was then rinsed with PBS for three times and developed with 3,3'-diaminobenzidine (#D5905; Sigma-Aldrich). The tissue was rinsed with 1X PBS for three times then mounted onto positively charged slides (EF15978Z, Daigger®). The mounted tissue sections were allowed to dry overnight at room temperature and dehydrated using ethanol series. Then, the sections were cover slipped using VectaMount® Permanent Mounting Medium (H-5000-60).
