# Supplementary material for: Selection of single domain anti-transferrin receptor antibodies for blood-brain barrier transcytosis using a neurotensin based assay and histological assessment of target engagement in a mouse model of Alzheimer’s related amyloid-beta pathology
Source: PLoS One. 2022 Oct 18;17(10):e0276107. doi: 10.1371/journal.pone.0276107 (PMC9578589; doi:10.1371/journal.pone.0276107)
Supplement: S1 Raw images — (PDF) [file pone.0276107.s016.pdf]

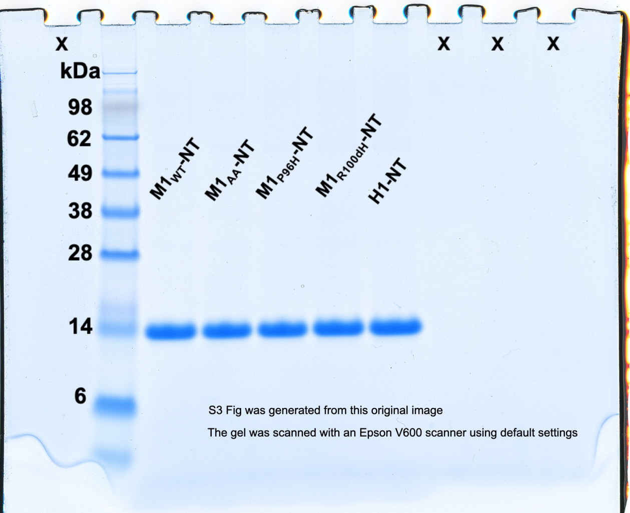
x
kDa
98
62
49
38
28
14
6
x
x
x
M1WT-NT
M1AA-NT
M1P96H-NT
M1R100dH-NT
H1-NT
S3 Fig was generated from this original image
The gel was scanned with an Epson V600 scanner using default settings

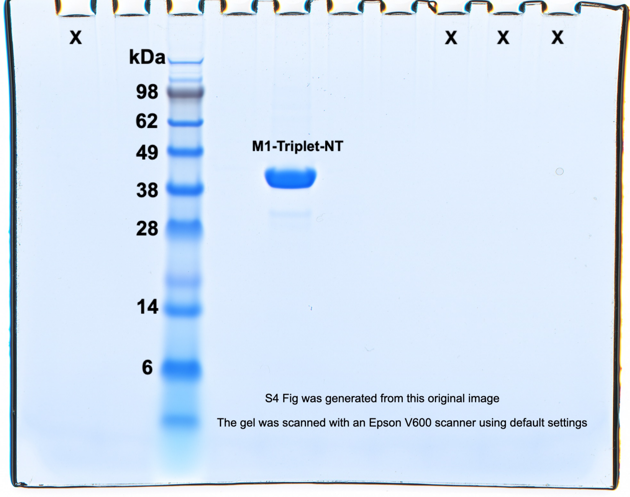
X
X
X
X
kDa
98
62
49
38
28
14
6
M1-Triplet-NT
S4 Fig was generated from this original image
The gel was scanned with an Epson V600 scanner using default settings
